# The human tubal lavage proteome reveals biological processes that may govern the pathology of hydrosalpinx

**DOI:** 10.1038/s41598-019-44962-1

**Published:** 2019-06-20

**Authors:** Elizabeth Yohannes, Avedis A. Kazanjian, Morgan E. Lindsay, Dennis T. Fujii, Nicholas Ieronimakis, Gregory E. Chow, Ronald D. Beesley, Ryan J. Heitmann, Richard O. Burney

**Affiliations:** 1 Department of Clinical Investigation, Division of Graduate Medical Education, Madigan Army Medical Center, 9040 Jackson Ave, Tacoma, WA 98431 USA; 2 Department of Obstetrics and Gynecology, Division of Reproductive Endocrinology and Infertility, Madigan Army Medical Center, 9040 Jackson Ave, Tacoma, WA 98431 USA

**Keywords:** High-throughput screening, Protein-protein interaction networks

## Abstract

Hydrosalpinx, the blockage of fallopian tubes, can result from pelvic inflammatory disease. Hydrosalpinx is a cause of infertility and negatively impacts *in vitro* fertilization. To better understand the pathobiology of hydrosalpinx, we compared the proteome of lavages from disease vs. healthy fallopian tubes. Results indicate a disruption of redox homeostasis and activation of the complement system, immune cell infiltration, and phagocytosis; pathways that may drive tubal injury. To our surprise among the most prominent proteins with hydrosalpinx was mesothelin (MSLN), which until now has only been associated with epithelial malignancies. Analogous to mesothelioma and ovarian carcinoma, a significant increase of MSLN was detected in plasma from patients with hydrosalpinx. This finding suggests MSLN may provide clinical diagnosis in lieu of the current approaches that require invasive imaging. Importantly, these findings implicate MSLN in a benign disease, indicating that the activation and role of MSLN is not restricted to cancer.

## Introduction

Hydrosalpinx is a condition wherein occluded fallopian tubes fill with fluid and become distended. Typically, hydrosalpinx is a complication of pelvic inflammatory disease (PID), which can be painful and negatively impact reproductive health^1^. Many women with PID are subclinical or asymptomatic, resulting in delayed or no treatment^2^. With diagnosis and treatment, PID can still result in infertility, ectopic pregnancy, and/or chronic pelvic pain. These adverse outcomes are compounded by the degree of tubal damage resulting from hydrosalpinx^3^.

Procedures for infertility generally include an assessment of tubal patency via hysterosalpingography and/or laparoscopy. Hydrosalpinx can be reliably detected with these diagnostic tests, however, they are expensive, invasive, and not without risk. A 2011 National Institutes of Health Workshop identified research needs related to the diagnosis, treatment, and prevention of PID. Among these research needs was a call to “identify biomarkers correlated with PID, its sequelae, as well as non-invasive tests to detect and measure them”^4^. However, a thorough molecular characterization remains a prerequisite to the development of biomarkers for hydrosalpinx.

Several studies have shown detrimental effect of hydrosalpinx on *in vitro* fertilization (IVF) success rates. Poor IVF outcomes with hydrosalpinx include, a 50% reduction in pregnancy and 2-fold increase in spontaneous abortion^5^. Surgical removal of hydrosalpinx or proximal tubal occlusion can improve IVF success^6^. Transvaginal aspiration of hydrosalpinx fluid can also improve the success of IVF^7^. Although these interventions can resolve hydrosalpinx, they are invasive, costly, and do not entirely correct reproduction. The absence of non-invasive treatments is related to our lack of understanding the molecular mechanisms that govern the pathogenies of hydrosalpinx.

Fallopian tube fluid is a complex mixture of components secreted from the epithelial cells and blood plasma to support early embryo development. Our understanding of the tubal fluid components largely stems from animal studies. Notably, the characterization of proteins in animal tubal fluid has contributed to the development of cleavage stage embryo culture media^8,9^. In contrast, little is known regarding the composition or the proteome of human tubal fluid partly due to the difficulty in accessing bio-specimens. A characterization of tubal fluid in healthy and diseased individuals may reveal the factors that promote tissue pathology and perturb fertility in hydrosalpinx.

To address this crucial gap, we have analyzed lavages from those afflicted with hydrosalpinx and healthy fallopian tubes using label-free shotgun proteomics^10^. Statistical analysis of protein abundances differences between normal and disease states was conducted using DESeq2 package (Bioconductor)^11^. Though those such packages (DESeq, edgeR, and baySeq) originally developed for RNA-seq, recently applied for proteomics count data^12,13^. Deferentially expressed proteins were then mapped onto regulatory pathways to identify those involved in disease. This analysis uncovered significantly dysregulated pathways and bioprocesses in hydrosalpinx. Importantly, we identified several proteins of interest that may play a role in the pathogenesis of hydrosalpinx. The most prominent proteins, including mesothelin (MSLN), were validated by immunoblotting and immunohistochemistry. With these integrated approaches, we have profiled the proteome of hydrosalpinx and identified potential indicators of tubal pathology.

## Results

**Figure 1:**
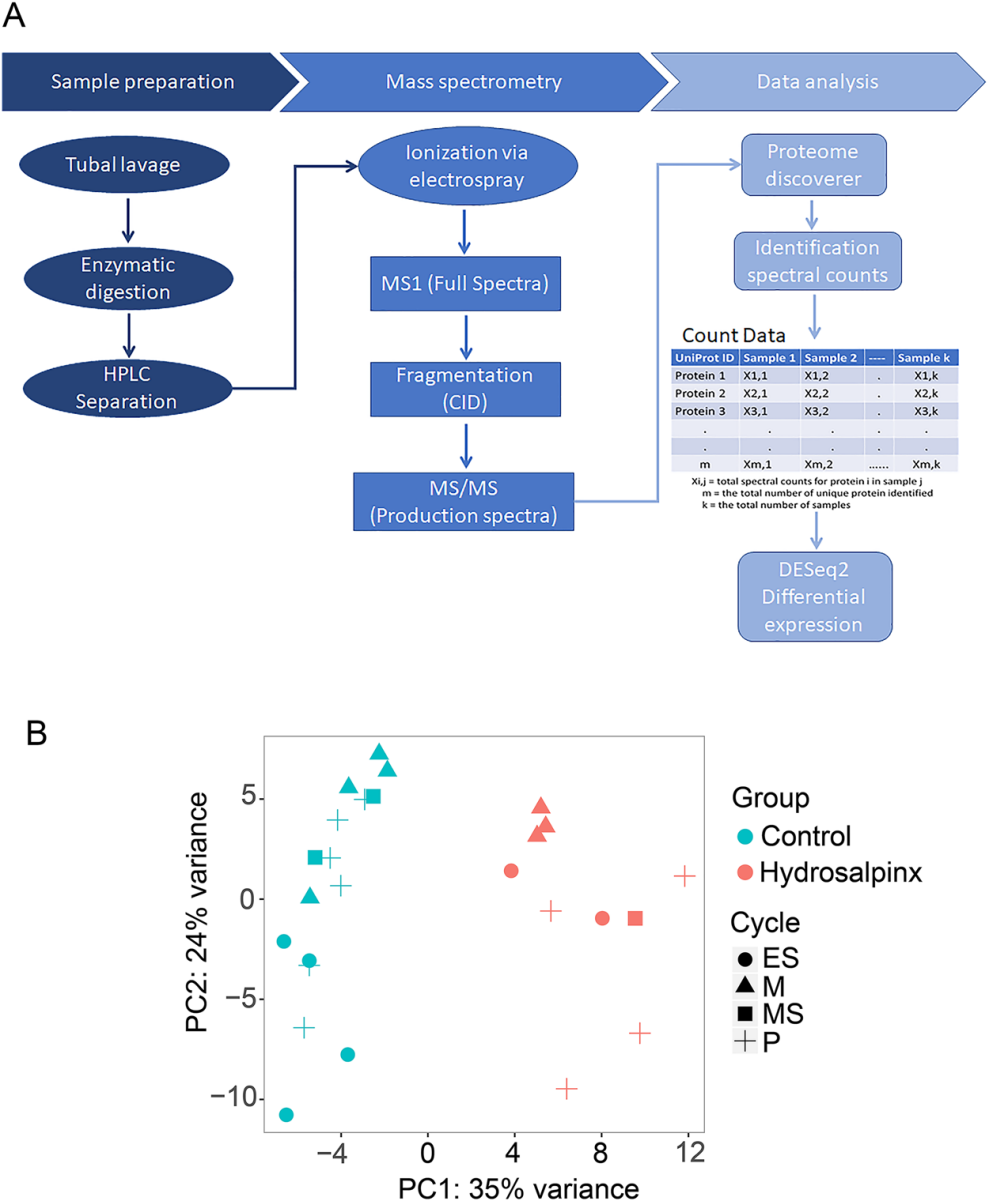
High-dimensional data summary. (**A**) Shotgun spectral counting proteomics workflow. (**B**) Principal components of samples: samples from subjects with tubal hydrosalpinx (red) and from subjects of healthy controls (blue). Data point shapes represent menstrual status: menstrual (M), proliferative (P), early secretory (ES), and mid-secretory (MS). Each plotted point represents an individual sample’s proteome expression profile distributed into a two-dimensional space based on the variance in proteome abundance. The axes represent the two principal components with the percentage of protein abundance variation explained by each component.

Label free LC-MS/MS analysis (Fig. 1A) provided coverage of over 5,000 peptides which collectively mapped to 519 non-redundant proteins within the two experimental groups. With respect to cellular localization, 47% of the 519 proteins were extracellular, the remainder were cytosolic (39%), nuclear (10%) and proteins with unknown localization (4%).

### Principal component analysis (PCA)

The relationship among samples was visualized by performing PCA on rlog transformed spectral count data (Fig. 1B). For the 519 proteins, the primary principal component distinguished 35% of the variance, with 24% additional variation shown by the second principal component. Samples clustered mainly by disease status (hydrosalpinx or healthy (control)) with no overlap. PCA indicates that the differences between experimental groups is more considerable than within group sample differences. Patterns corresponding to the experimental groups also emerge from the heatmap (Fig. S1A). In this case, it is clear that the expression vectors (the columns of the heatmap) for samples within the same cluster coincide with either disease or control phenotypes.

### Differentially expressed proteins

Once the normal p-value distribution was evident (Fig. S1B), p-values were adjusted to control for false discovery rate. Proteins considered significant were with LogFC ≥ 1.5 or ≤−1.5 and adjusted p-value ≤ 0.05 per our power analysis result (Fig. S2). The fold changes and p-values for 116 proteins that passed these criteria were provided in Table S2 and highlighted as red dots in Fig. S1C. Among these 116 proteins, 76 were up-regulated and 40 were down-regulated with hydrosalpinx. Inferences for these differentially expressed proteins including peptide sequences, identification scores, protein scores and coverages, number of protein groups and Uniprot accessions are provided in the database search result (Table S3).

### IPA core analysis

**Figure 2:**
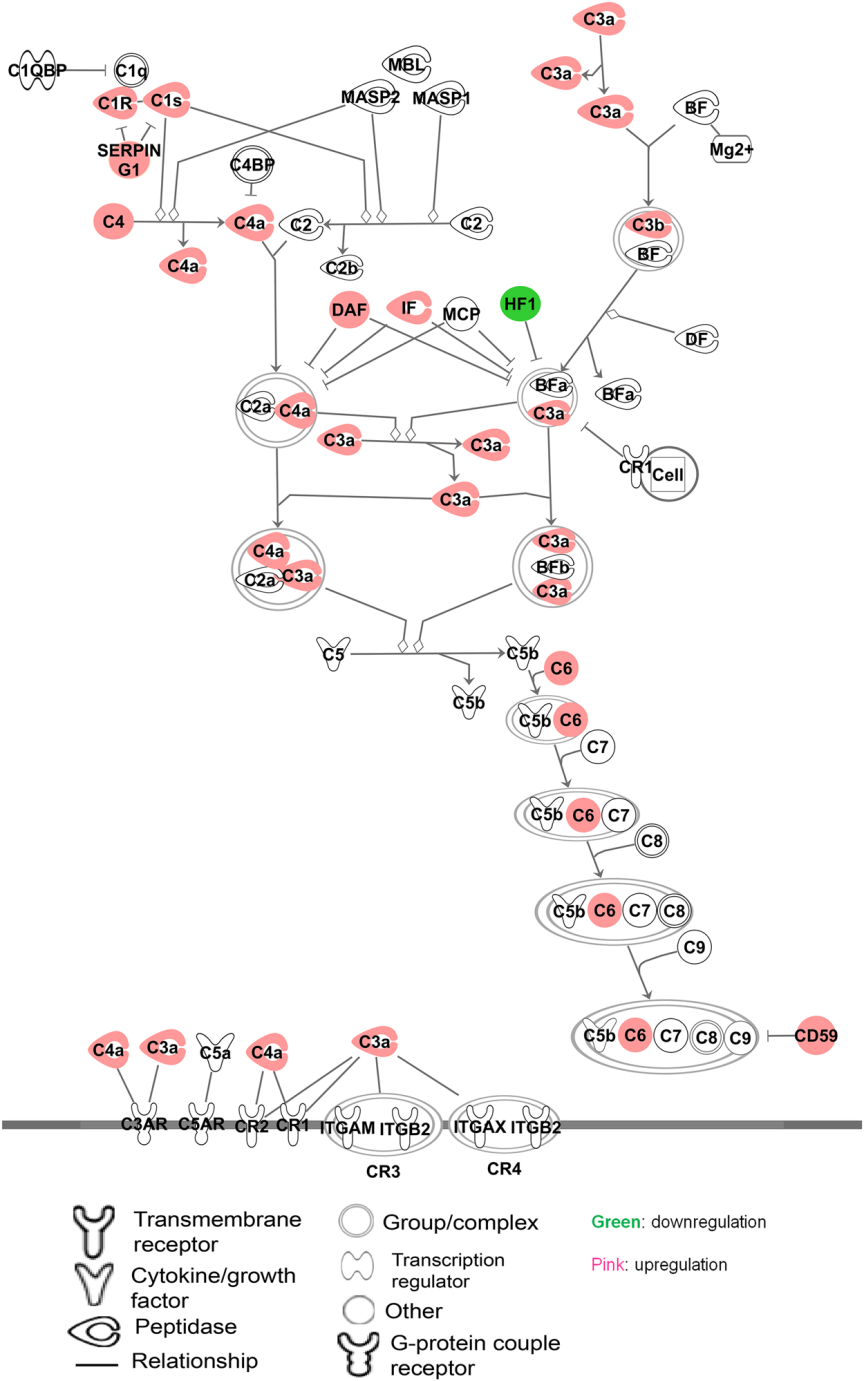
Complement system. A pre-defined pathway showing significant enrichment of associated proteins in hydrosalpinx. On this pathway, the proteins from this study are highlighted with pink (up-regulated) and with green (down-regulated) in hydrosalpinx. Legend for the shapes of molecules and relationship on the pathway are shown at the bottom. The pathway was generated through the use of IPA (QIAGEN Inc., https://www.qiagenbioinformatics.com/products/ingenuity-pathway-analysis)^14^.

**Figure 3:**
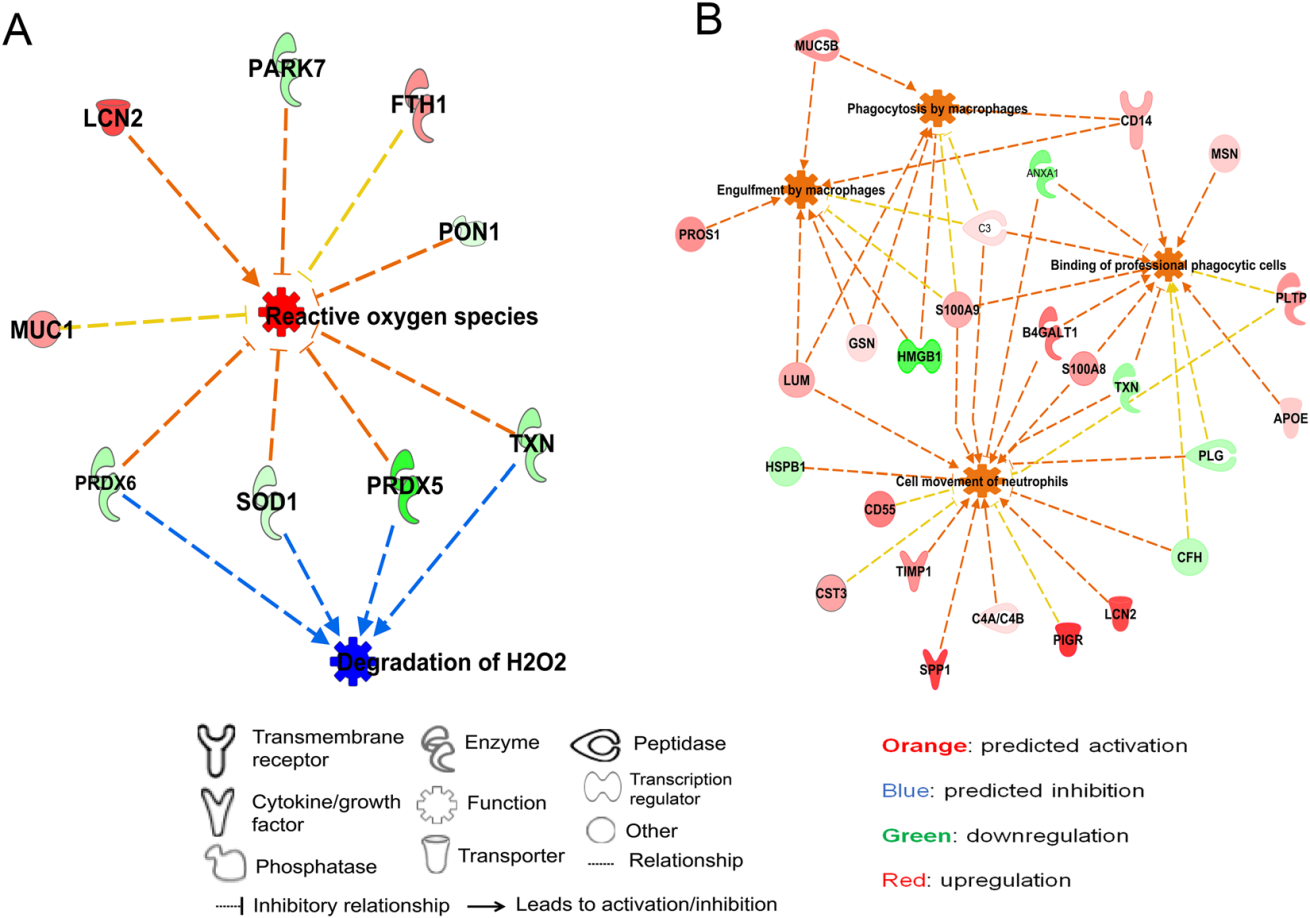
Networks of protein modules: (**A**) Proteins networks involved in the activation of ROS and reduction of hydrogen peroxide degradation. (**B**) Protein networks that are involved in the immune cell infiltration and activation of phagocytosis. In these two networks of protein modules, bioprocesses in the center are represented by nodes highlighted with orange (activated) and blue (deactivated) in hydrosalpinx. Legends at the bottom denote the representation of molecules shapes, highlights and their relationship to each networks. The networks were generated through the use of IPA (QIAGEN Inc., https://www.qiagenbioinformatics.com/products/ingenuity-pathway-analysis)^14^.

The relationship of the differentially expressed proteins and their enrichment in pre-defined pathways were generated through the use of IPA (QIAGEN Inc., https://www.qiagenbioinformatics.com/products/ingenuity-pathway-analysis)^14^. The significant results from this analysis are summarized in the pre-defined pathway (Fig. 2) and in the form of networks (Fig. 3). Enrichment analysis revealed that 25% of the proteins up-regulated in hydrosalpinx are linked to the complement pathway (Fig. 2).

There was also an increase of proteins related to reactive oxygen species (ROS) generation (Z-score 2.44; p-value = 6.79E-06) and decrease in proteins that metabolize hydrogen peroxide (Z-score −2.16; p-value = 7.96E-08) (Fig. 3A). This association was consistent with the directionality of the fold changes for the proteins on this network with the exception of mucin 1 (MUC1) and ferritin heavy chain 1 (FTH1). Proteins involved in activation of immune cell trafficking, inflammation and macrophage phagocytosis were also up-regulated in hydrosalpinx (Fig. 3B). Conversely, proteins related to the inhibition of these biological processes were down-regulated (Fig. 3B).

### Orthogonal validation by immunoblotting

**Figure 4:**
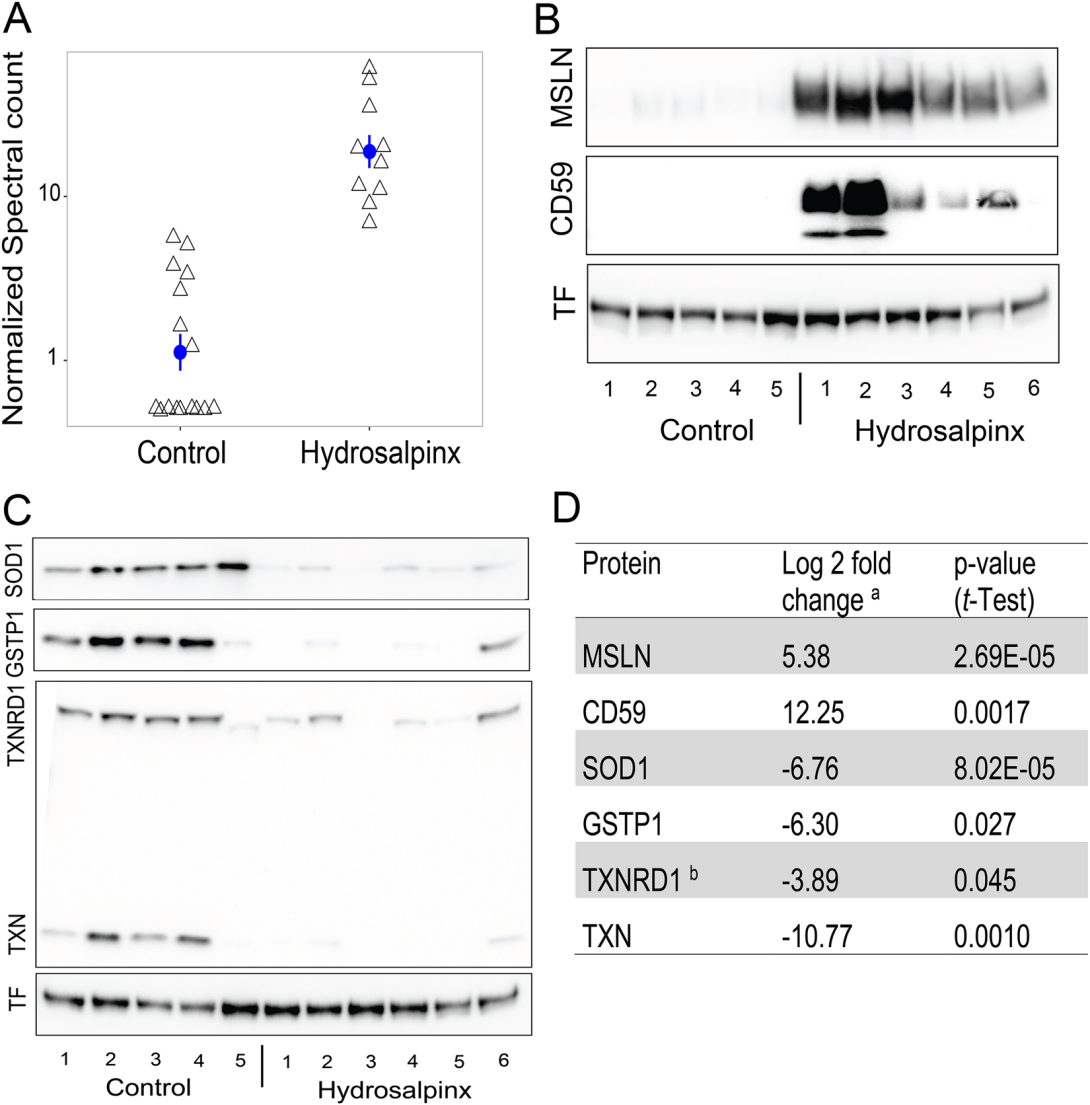
Protein abundance for selected proteins. (**A**) Scatter plot of normalized spectral counts for MSLN in control (n = 16) and hyrdosalpinx (n = 10) lavages. Mean and SEM are represented with a blue circle and error bar respectively. (**B**) Immunoblot analysis for MSLN, CD59, and loading control Transferrin (TF). **(C)** Immunoblot analysis for SOD1, GSTP1, TXNRD1, TXN and TF loading control. See supplemental Fig. S5 for the full length blot images for each protein target. (**D**) Table of relative fold changes for the proteins represented in panels B and C. ^a^The log fold change is expressed as the log ratio between the mean intensity of immunoblot for the hydrosalpinx relative to control after normalization to TF. ^b^Protein identified via IPA network analysis represented in Fig. 3.

Immunoblot analysis was conducted to verify the directionality of fold changes for the most prominent proteins identified by shotgun spectral counting. A subset of 11 patient samples (six hydrosalpinx and five healthy tubal lavages) were analyzed for MSLN, CD59 and proteins that are involved in oxidative stress pathway including peroxiredoxins (PRX) SOD1, GSTP1, TXN and TXNRD1 (Fig. 4). MSLN and CD59 were among the most overabundant proteins in hydrosalpinx fluid. Scatter plot of MSLN expression levels stratified by cohort revealed no overlap in expression between hydrosalpinx and control specimens (Fig. 4A). Immunoblot analysis verified the abundance of MSLN and CD59 in hydrosalpinx (Fig. 4B). In contrast, SOD1, GSTP1, TXN and TXNRD1 were lower with hydrosalpinx (Fig. 4C). Quantification of the immunoblot analysis supports the significant difference in the relative fold changes of these proteins between experimental groups (Fig. 4D).

Immunoblot analysis revealed that MSLN and CD59 run as broad bands, well over and below the expected molecular weights 40Kda and 18Kda respectively (Fig. 4B). Both MSLN and CD59 are glycosylphosphatidylinositol (GPI)-anchored glycoproteins^15–17^. It is possible that these samples have multiple isoforms of these proteins due to post-translational modifications^18^. N-linked glycosylation variances analysis using gel shift assay (Fig. S3) validated that the bands for both MSLN and CD59 in the original samples without de-glycosylation represent mature and various degree of N-linked glycosylated versions of the two proteins. The three sharp bands after de-glycosylation suggests the presence of additional species for MSLN.

The result from IPA core analysis shown in Fig. 3A delineated alterations in PRX protein abundance. Consistent with this finding, immunoblot analysis for key enzymes using the PRX pathway immunoblot cocktail (Abcam) revealed that hydrosalpinx contained about 11- and 4- fold less TXN and TXNRD1 respectively, compared to healthy lavages (Fig. 4C,D). However, the analysis did not show any signal for PRX1. In addition, verification immunoblot analysis for additional redox modulator proteins including GSTP1 and SOD1 delineated significant suppression of both GTP1 and SOD1 in hydrosalpinx compared to healthy control (Fig. 4C,D).

### Immunohistochemistry

**Figure 5:**
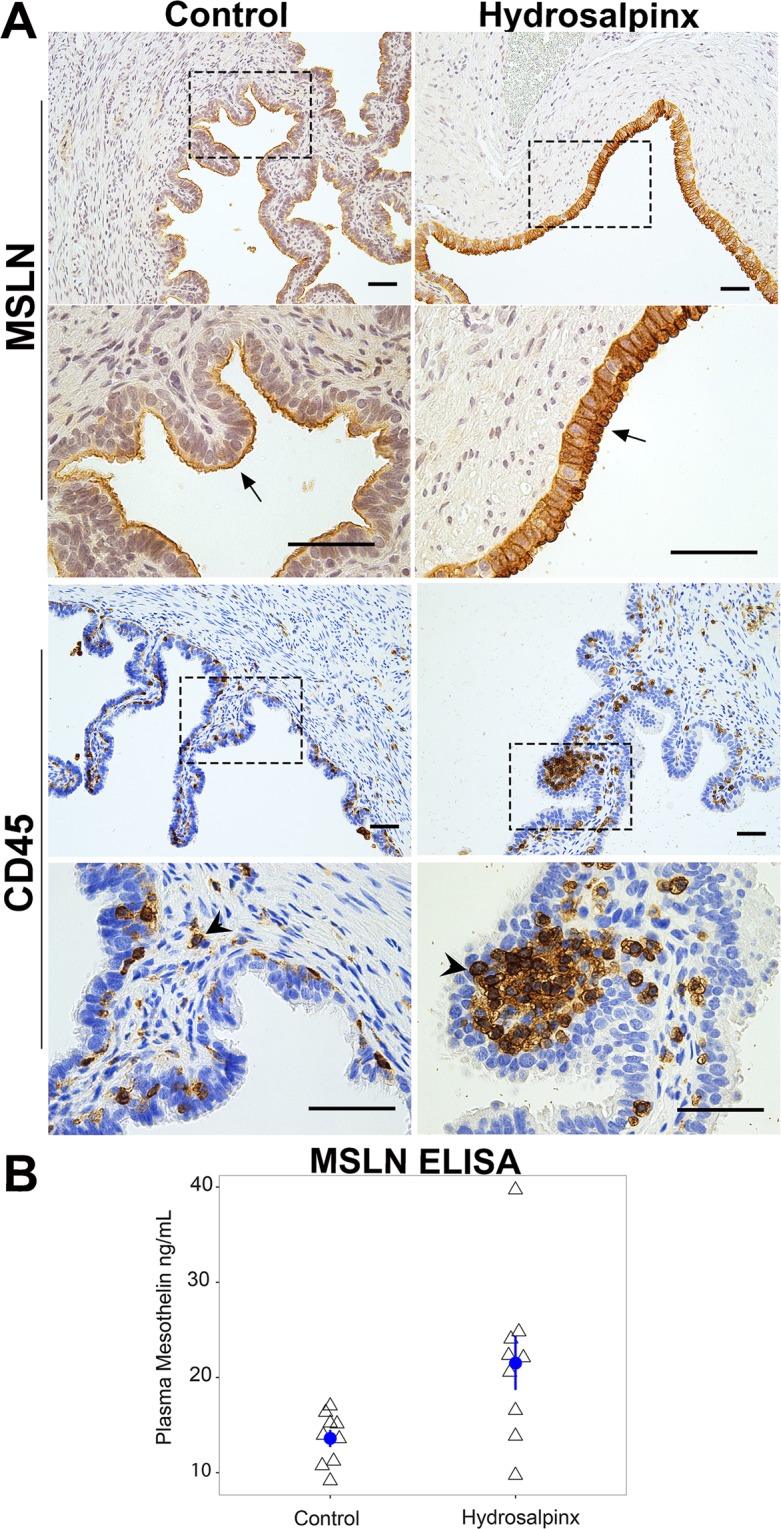
MSLN is concentrated to the epithelium and elevated in the plasma of women with hydrosalpinx. (**A**) MSLN and CD45 staining in cross-sections from healthy control (left column) and hydrosalpinx afflicted fallopian tubes (right column). Black perforated boxes in the top rows for MSLN and CD45 indicate the region where higher magnification (scale bar 500 µm) are represented in the bottom rows. Arrows and arrows heads indicate where MSLN and CD45 positive cells are concentrated. (**B**) Scatter plot of plasma MSLN in control (n = 9) and in women with hydrosalpinx (n = 9) assayed by ELISA. Mean and SEM for each group are represented with blue circle and error bar respectively.

To gain insight on the localization of MSLN, immunohistochemistry (IHC) analysis was carried out in fallopian tissue sections from hydrosalpinx and healthy donors. MSLN staining appears more prevalent in the mucosa epithelium with hydrosalpinx. Mucosa folds seen in the healthy fallopian tube mostly disappear in hydrosalpinx, leaving behind a hollow and distended tube. Staining for CD45, reveals few immune cells within healthy tubal tissue (Fig. 5A). In contrast, CD45+ cells localized to the basal side of columnar epithelium appear more abundant within the mucosa of hydrosalpinx tissue (Fig. 5A). As predicted, there was no presence of CD45+ cells within the epithelium where MSLN staining was concentrated^19^.

### Plasma MSLN levels in subjects with hydrosalpinx and healthy fertile control

To determine whether MSLN is also elevated in peripheral blood, we analyzed plasma collected from women with and without hydrosalpinx by ELISA assay. As depicted in Fig. S4A, MSLN ELISA assay was linear for sample with highest MSLN concentration. The mean plasma concentration of MSLN in women with hydrosalpinx (21.52 ± 2.81 ng/mL) was significantly higher vs. healthy controls (13.60 ± 0.89 ng/mL, p < 0.05) (Fig. 5B). Of the nine women with hydrosalpinx who had elevated MSLN in the tubal fluid (Fig. 4A) six had higher plasma MSLN.

## Discussion

Adverse outcomes associated with hydrosalpinx have been well documented^4,20–23^. To gain insight on the processes that govern the pathology of hydrosalpinx, we characterized the proteome of fallopian tube lavage. We reasoned that secreted or shed proteins would indicate the underlying mechanism of tissue injury^24^. Our analysis identified a total of 519 proteins in the healthy lavage and hydrolapinx fluid combined using a label free shotgun proteomics platform. We utilized DESeq2 to quantify the relative abundance of these proteins between normal and disease states. DESeq2 is one of the three widely used open sours packages for RNA-seq count data^25,26^. The statistical approaches adapted in these packages are powerful as they addresses the issue of the appropriate probability distribution for count data as well as tackles the paucity of information due to the absence of enough replicate samples. These packages have become powerful statistical alternatives for the proteomics community to perform differential expression analysis of spectral count data^12,13^. Using DESeq2 package, we report changes in abundance for over 110 proteins in hydrosalpinx fluids compared to lavages of healthy fallopian tubes.

### MSLN and binding partners are over-abundant in hydrosalpinx

Among over-abundant proteins in hydrosalpinx were MSLN and binding partners MUC1, mucin 5B (MUC5B), and mucin 16 (MUC16), from which the antigen CA125 is used to diagnose ovarian cancer^27^. MSLN expression and its distribution in normal human tissue is limited to the mesothelial cells lining of the pleura, peritoneum, and pericardium^28^. However, elevated levels of MSLN are highly correlated with mucins in several types of human cancers^29–31^. In many cancers, MSLN is actively shed from cell surface, generating an antigen pool in the circulation and the tumor interstitial space^32,33^. Analogous to finding in cancer, we also observed increase in both bound and soluble MSLN in women with hydrosalpinx (Fig. 4A,B top panel and D, Fig. 5A top panel, Table S2). In addition, a significant increases of MSLN was observed in plasma from women with hydrosalpinx (Fig. 5B).

MSLN is a C-terminal cleaved product of the parent 69Kda protein encoded by MSLN gene. This cleavage also gives rise to N-terminal 31Kda megakaryocyte potentiating factor (MPF). LC-MS/MS analysis of in-gel digest for the before and after deglycosylated samples identified peptide sequences spanning the N- to the C-terminus of the parent 69Kda protein. However, there was no band around 69Kda precursor on the immunoblot (Fig. S3) suggesting that the N-terminus peptides were not from the 69Kda parent protein but were enzymatic digests of MPF. These results demonstrate the presence of both MSLN and MPF in hydrosalpinx; a combination documented in cancer pathology^29,34,35^.

Unlike MSLN, the different isoforms of mucins are ubiquitous and they are produced by epithelial tissues. Expression of cell-surface and gel-forming mucins can be up-regulated by inflammatory cytokines such as interleukins, interferons, and tumor necrosis factor alpha^36–38^. It has been shown that neutrophils stimulate production of both gel-forming and cell-surface mucins by mucosal epithelial cells^39–41^. These reports and the results from our study support a link between mucins, innate mucosal immunity, and the mucosal inflammatory response.

The overexpression of MSLN and mucins in many adenocarcinomas has been implicated in cell adherence, cell survival/proliferation, tumor progression, and chemo-resistance^15,42^. The biological function of MSLN is still largely unknown. The deletion of *Msln* in mice results in no obvious developmental or postnatal phenotypes^43^. Despite these gaps, the upregulation of MSLN mainly in cancers makes for an appealing therapeutic target^44^. Several pre-clinical and phase I/II clinical trials are currently evaluating antibodies against MSLN and mucins^45–48^.

The overabundances of MSLN and mucins with hydrosalpinx has not been reported previously and warrants further investigation. In addition, the overlapping pathophysiology involving MSNL between cancer and hydrosalpinx is yet to be determined. However, this result demonstrates that hydrosalpinx shares molecular targets with tubal and/or ovarian cancer pathology. It is possible that MSLN overexpression with hydrosalpinx precedes tubal and/or ovarian cancer. Alternatively, MSLN is related to the disease process of hydrosalpinx that is unconnected to the malignant transformation of reproductive tissue. In this case it would be the first instance of MSLN overexpression in the context of a disease process unrelated to cancer.

MSLN in the sera of patients with ovarian cancer and mesothelioma has been identified as diagnostic biomarker^33,35^. Analogues to ovarian cancer we also observed a significant increase of MSLN in plasma from patients with hydrosalpinx. Hydrosalpinx typically presents in sexually active woman at an average age of 25 years^49^. Ovarian cancer occurs predominately in woman middle age or older with a family history of cancer^50^. It is possible that MSLN has utility as a non-invasive diagnostic biomarker for hydrosalpinx in younger woman with prior history of PID. However, it would not be able to distinguish hydrosalpinx in woman at risk for ovarian cancer. The other limitation worth noting, is that not all woman with higher concentrations of MSLN in tubal lavages exhibited greater amounts in their plasma (Fig. 5B). Similar heterogeneity in plasma MSLN concentrations among patients with mesothelioma and ovarian carcinoma have partly been attributed to the lack of MSLN expression in their tumors, and/or to differences in the study population^51^. A larger cohort of patients is necessary for determining the diagnostic accuracy of MSLN for hydrosalpinx and addressing the mechanisms of its absence in subset of patients that are afflicted.

### Inflammatory protein modules are overabundant in hydrosalpinx

Nearly two third of the up-regulated proteins in hydrosalpinx are associated with inflammation. Subsets of these proteins are involved in neutrophils taxis and infiltration, binding of phagocytic cells, and phagocytosis by macrophages. The identification of these processes suggest that the inflammation with hydrosalpinx involved myeloid cell trafficking and activity. The other subset of proteins are related to the complement system. Among those implicated are, 10 out of 37 known complement components and regulators including CD55 and CD59. The overexpression of over 25% of the complement proteins, via enrichment analysis, indicates that the complement system plays a substantial role in the pathology of hydrosalpinx (Fig. 2).

In the past few decades evidences have shown that complement systems have much broader functions in immune surveillance and homeostasis^52^. Complement assists in the clearance of immune complexes, cellular debris and apoptotic cells, and it has been associated with early development and tissue repair^53^. On the contrary, complement is also known to be pro-inflammatory and to cause necrotic cell death^54^. The balance between anti-inflammatory properties and pro-inflammatory actions of complement maybe a critical determinant of tissue restoration or pathology. Whether the over-abundance of complement proteins is protective or damaging in the context of hydrosalpinx pathology is yet to be determined.

It is known that self-cells are normally protected from complement by membrane bound complement regulators including CD46, CD55 and CD59. *In vitro* experiments indicate that the loss of both CD46 and CD59 on the cell surface of neutrophils undergoing apoptosis, leads to their susceptibility for complement-mediated lysis^55^. This process coincides with the accumulation of CD46 and CD59 in the supernatant. Similarly, overabundance of both CD55 and CD59 in hydrosalpinx fluid may have resulted from loss of these proteins on the cell surface. In addition, the soluble form of CD59 detected in urine has shown to retain its specific complement binding activity but exhibits greatly reduced ability to inhibit complement membrane attack^17^.

The accumulation of soluble CD55 and CD59 coupled with an increase in complement-associated proteins suggest that complement mediated lytic processes may contribute to the tissue damage observed in tubes affected with hydrosalpinx^56^. Importantly, a recent study also suggest that complement is not a strictly intravascular system; instead, local secretion of complement components by tissue and infiltrating cells, and potentially even intracellular complement turnover, contribute to the overall complement response in many circumstances^57,58^. In this regard, overabundant complements in the hydrosalpinx may have been from local secretion. Taking together, our results suggest that complement activation can be one of the mechanism by which hydrosalpinx orchestrate chronic inflammatory process, the different degrees of tubal mucosa pathology.

### Dysregulated protein modules in hydrosalpinx

Among the dysregulated proteins in hydrosalpinx fluid, relative to lavages from healthy donors, were detoxification enzymes involved in the ROS defense mechanism. This down-regulation may result in lower hydrogen peroxide clearance during respiration and greater susceptibility to oxidative damage from the generation of ROS by proinflammatory cells (Fig. 3A). Oxidative stress is a central feature of a number of inflammatory processes associated with infertility^59,60^ and may explain the embryotoxicity of hydrosalpingeal fluid^61^.

The expression and activity of detoxification enzymes within the fallopian tube is critical for normal reproductive physiology. Intratubal expression of SOD1, GSTP1, TXN and PRX1 are induced by the presence of gametes in the oviduct and important for successful fertilization, embryo cleavage and/or embryo transit^9,62^. For sperm viability and motility, SOD1 is routinely added to sperm cryopreservation media^63,64^. Furthermore, the addition of antioxidant enzymes such as SOD1 and catalase (CAT) to oocyte *in vitro* maturation media improves fertilization and embryonic cleavage rates^65^. In contrast, the addition of ROS such as xanthine and xanthine oxidase decreases both fertilization and embryonic cleavage rates^65^.

Consistent with these preclinical observations, we noted the presence of SOD1 in lavages from healthy fallopian tubes irrespective of menstrual cycle phase (Fig. 4C). In hydrosalpinx fluid we observed a significant decline of key redox pathway enzymes to include SOD1, GSTP1, TXN, PRDX5, and PRDX6. The down-regulation of these key detoxification enzymes may result in oxidative stress, detrimental to both gamete viability and early embryo development. Collectively, these findings and those of others^61,66^, support increased inflammation and oxidative stress as the molecular basis for the observed embryotoxicity and impairment of embryonic implantation with IVF.

Proteins associated with protection from ROS mediated cell death were also decreased in the hydrosalpinx fluid compared to fluid from healthy fallopian tubes. ROS alter most cellular molecules such as lipids, proteins and nucleic acids. If not reduced by enzymatic or non-enzymatic antioxidation pathways, ROS lead to cell death. In hydrosalpinx fluid, we observed decreased amounts of selenium binding protein 1 (SELENBP1), aldolase (ALDOA), protein/nucleic acid deglycase DJ-1 (PARK7), heat shock protein family members (HSPB1, HSPA2) and glyceraldehyde 3-phosphate dehydrogenase (GAPDH). These proteins mediate protection against stress, cell death and tissue necrosis^67–69^. HSPB1 prevents apoptosis via directly inhibiting caspases (CASP9 and CASP3) and dysregulation of HSPB1 results in cell death^70^. In addition to its function in glycolysis, GAPDH has an important role in DNA repair and replication, post-transcriptional regulation, gene expression and cell death^71,72^. The decline of proteins in the two interrelated processes, cell protection and antioxidant, support the involvement of oxidative stress in the pathophysiology of hydrosalpinx.

Strengths of this study include the collection of tubal fluid from microtubal anastomosis surgeries and its proteome characterization. A limitation of the study is the potential for proteomics results being confounded by blood contaminant, particularly in lavage specimens. To minimize this, tubal lavages were performed with gentle infusion on a saline rinsed surgical field and exclusion of specimens that showed visible signs of blood contaminants. Another limitation is our proteome analysis may not have reliably surveyed low abundant proteins as spectral counting inherently skews toward the identification of highly abundant proteins. Nonetheless, this work demonstrates the importance of using a shotgun spectral counting technique for analyzing proteome abundance.

It worth noting that DESeq2 is based on negative binomial variance modeling with the assumptions: (1) that the data are roughly count scale and (2) the observations (spectral counts) can be assigned to proteins with some certainty. The second assumption fails when proteins with significant homology are largely represented in the dataset, and/or databases used for identification are redundant and contain several nearly-identical sequences. To alleviate this problem we utilized the principles of parsimony, assigning peptides to proteins that correspond with maximum number of identified peptides. We also utilized a well curated protein sequence database with a minimal level of redundancy. Finally, the proteomics analysis is supported by orthogonal validation. Nonetheless, this report is among the few that have applied DESeq2 for differential analysis of spectral counts from proteomics data^12^. Therefore, further examination and validation of DESeq2 within the context of proteomics analysis, would provide greater confidence for this application.

In summary, our results broaden and enrich our understanding of the proteins that are secreted or shed in healthy and hydrosalpinx afflicted fallopian tubes. Importantly the identification of MSLN in hydrosalpinx is a novel finding that may provide diagnostic value. Finally, our results implicate the complement pathway and oxidative stress as potential molecular mechanisms for mucosal fold damage and embryotoxicity that occur with hydrosalpinx.

## Methods

### Patient selection and clinical samples

Study approval was granted by the Madigan Army Medical Center Institutional Review Board (Protocol No. 212093). Volunteer women age 18–45 have been used as the source of material for the described work outlined in this manuscript. Subjects in the normal group (Table S1) were fertile with a history of isthmic tubal interruption and desired fertility requesting microtubal anastomosis (MTA) surgery. Prior to laparotomy for MTA, a laparoscopic survey was conducted to assess adequacy of segments for re-anastomosis at which time a normal pelvis (no endometriosis, tubal disease or leiomyomata) was visually documented. Whereas women in the hydrosalpinx group (Table S1) were identified as having a communicating hydrosalpinx by hysterosalpingogram (HSG) performed three or more months prior to surgery. At laparoscopy, hydrosalpinx was further confirmed by the presence of tubal distention >3 cm diameter in the setting of distal tubal phimosis and a variable degree surrounding pelvic and/or perihepatic adhesions.

Once written informed consent provided by the study participants (see Table S1- for details in subjects’ demography), sera, plasma, and fallopian tube aspirates from women with and without hydrosalpinx were collected according to the IRB guidelines and regulation. Briefly, prior to general anesthesia, serum was collected by venipuncture for estradiol and progesterone analysis to assess the menstrual state of each participant. Lavages from healthy donors were irrigated with 1 mL saline from the proximal end and collected from the distal fimbriated end of the tube in a sterile conical tubes before the initiation of microtubal anastomosis surgery. Hydrosalpinx specimens were collected from the isthmic-ampullary region using a laparoscopic aspirating needle attached to a sterile 10 cc syringe prior to salpingectomy for hydrosalpinx-associated infertility. All the specimens were transferred into sterile collection tubes, immediately placed on ice, and taken to the lab for further processing.

### Experimental design and statistical rationale

#### Sample size and power analysis

To estimate the number of samples a power analysis was performed using a pilot shotgun proteomics data set and an open source RnaSeqSampleSize package (http://www.biocondactor.org). The within-group median dispersion (variances) was computed using DEseq2 ((http://www.biocondactor.org) for a pilot spectral count data from hyrosalpinx fluids (n = 5) and lavages from normal patients (n = 7). The power curves per hypothesis of the negative binomial distribution test were generated as a function of sample size required to detect log2 fold changes of 1.5, 2, 3, at the adjusted p-value of 0.05, 0.01, and 0.001 and shown in Fig. S2.

### Label-free shotgun LC-MS

Cellular debris was cleared from tubal fluids immediately on collection by double centrifugation first to pellet and remove cells (200 g, 10 min, 4 °C) then to clear debris (14000 g, 10 min, 4 °C). The resulting supernatant was spiked with protease inhibitors (Halt protease inhibitor cocktail, 100×, Thermo Fisher Scientific) and stored at −80 until used for the downstream analysis. The general workflow shown in Fig. 1A. Briefly, each tubal fluid was normalized via BCA assay (Thermo Fisher Scientific), and diluted to 250 ng/µL. 5 µg of protein reduced and alkylated with DTT (12 mM) and iodoacetamide (36 mM) respectively. Proteolytic digestion was performed with modified trypsin (Promega) for 18 hours at 37 °C. A primary stock solution of Pierce peptide retention time calibration mixture standard (Thermo Fisher Scientific) containing heavy labeled lysine and arginine was prepared to a concentration of 1pmole/µL each. The standard peptides mix was added to each proteolytic digest to final concentration of 75 fmole/µL. Subsequently, about 600 ng of each digest was injected and separated by reverse-phase chromatography on 15 cm column (inner diameter 75 µm, 1.7 µm Acquity UPLC M-class peptide BEH C18) over 180 min gradient of 5 to 30% buffer B (0.1% [v/v] formic acid in 100% acetonitrile) using Acquity UPLC system (Waters). The LC system was directly coupled online with LTQ OrbitrapXL mass spectrometry (Thermo Fisher Scientific) via a nano-electrospray source. Mass spectra were acquired in a data-dependent manner with automatic switching between MS and MS/MS scans. Survey data was acquired from m/z of 380 to 1800 and each full MS scan was followed by six MS/MS scans using data-dependent acquisition with the dynamic exclusion option specified as follows: repeat count, 2; repeat duration, 30 s; exclusion duration, 45 s.

### Protein identification and data search parameters

The raw data files of interest were imported into Proteome Discoverer version 1.4 (Thermo Fisher Scientific) and peak lists were extracted using spectrum selector algorithm (general workflow shown in Fig. 1A). The resulting peak list files were then searched against the Swiss-Prot.fasta human database 20,183 sequences that was downloaded (March 23, 2017) from UniProt (http://www.uniprot.org/) to the local server and appended with decoy sequences using SEQUEST search engine. The search parameters applied to database searches included: (1) limiting the search to b/y ion, (2) tryptic peptides with up to 2 missed cleavages (C-Term K/R restrict P), (3) static modifications with carbamidomethyl (C), (4) dynamic modifications with deamidation (N/Q), and/or oxidation (M). Precursor and fragment mass tolerance was set to 10 ppm and 0.5 Da, respectively. Peptides identification were validated via running decoy database search with Percolator. Peptide-spectrum match (PSM) is considered correct if it achieved the estimated q-value (minimal false discovery rate) of 0.01 or less. For protein identification, a minimum of two peptides with delta Cn (delta correlation) ≤0.05 and with high confidence based on q ≤ 0.01 were utilized to ensure the protein level stringency. Peptides were grouped by both mass and sequence similarities. Protein were grouped via applying strict maximum parsimony principle.

### Quantitation and statistical analysis

The relative abundance for identified proteins was measured on the basis of the spectral count (the total number of identified peptide spectra matched to the protein of interest, including those redundantly identified). The spectral counts or PSMs and associated protein identity was exported to MS-Excel. This MS-Excel file was then processed to generate spectral count matrix (Fig. 1A). The count matrix cell in the i^th^ row and the j^th^ column indicates how many peptide spectra matched to protein i in sample j (which stems from an independent biological replicate). Missing count values were replaced with zero and zero counts in DESeq algorithm^11^ treated as some positive value below 1. The spectral count matrix along with the metadata Table, which contains samples ID, Factors and Levels were read into R.

For differential expression analysis, the raw count data was processed using an DESeq2 which is implemented as a package for the R statistical environment^11^ and available through Bioconductor repository (http://www.biocondactor.org). DESeq2 is an open source and one of the three widely used differential expression analysis methods for high-throughput RNA-Seq data and effectively applied to detect differential protein expression in label-free spectral counting proteomics^12^. The method and the workflow used to test for differential expression is described in detail by Love *et al*.^11^. Briefly, the analysis with DEseq2 started with the observed spectral count matrix where the matrix entries X*ij* indicate the number of spectral counts that have been unambiguously mapped to a protein “i” in a sample “j”. In case where there were multiple isoforms in the sample and they were resolved based on additional unique peptides, PSMs from the common peptides were shared among the isoforms. DESeq2 analysis is based on the assumption that X*ij* are observation from the negative binomial distribution with expected mean µ*ij* and dispersion α*ij*, the expected spectral count matrix is expressed as generalized linear model (GLM) of binomial family with log link^73^. Based on prior studies demonstrating sex-steroid regulation of protein expression in the fallopian tube^74,75^, we sought to control for menstrual cycle phase in comparing the proteomics signatures from hydrosalpinx-affected and normal tubes. Experimental samples from each group were matched for menstrual cycle phase (Table S1), and cycle phase was added to the model formulae as a second factor affecting protein expression. The raw spectral count and the metadata data were used to generate a DESeqDataSet object using DESeqDataSetFromMatrix. The DEseq function was run using DESeqDataSet object which sequentially performs estimate size factors, estimate dispersions, and negative binomial Wald Test analysis that are wrapped into a single DEseq function. Proteome result Table including log2 fold changes, p-values, and adjusted p-values were extracted using results function. Differential expressed proteins were then filtered with Benjamini Hochberg adjusted p-value ≤ 0.05 and summarized in Table S2.

### High-dimensional data visualization

The count data was first normalized and transformed to log2 scale using rlog. Principal components of samples and heatmap of the count Table were then visualized by performing PCA (plotPCA’ Bioconductor package) and heatmap analysis (pheatmap, Bioconductor package) respectively.

### Enrichment analysis

Cellular locations and biological insights for the proteins with significant changes (adjusted p-value ≤ 0.05) were generated through the use of IPA (QIAGEN Inc., https://www.qiagenbioinformatics.com/products/ingenuitypathway-analysis)^14^.

### Immunoblotting analysis

Tubal fluid collected from women with hydrosalpinx (n = 6) and tubal lavages from cycle phase matched healthy fertile women (n = 5) were processed for immunoblot analysis using standard procedures. Briefly, lavage samples were suspended in loading buffer containing SDS and reduced with DTT. Twenty micrograms of total proteins from each sample were resolved on 4–12% polyacrylamide gels (Invitrogen) and transferred onto nitrocellulose membrane. The membrane was blocked with 5% skim milk, washed, and incubated overnight with specific primary antibody against MSLN (0.59 ng/µl), CD59 (8 pg/µl) SOD1 (1.11 ng/μl), GSTP1 (0.04 ng/µl), PRX pathway (cocktail of thioredoxin-1 (TXN), peroxiredoxin-1 (PRX1), and thioredoxin reductase-1 (TXNRD1), at 0.38×/μl), or TF (0.70 ng/µl). MSLN and CD59 are rabbit monoclonal IgGs from Cell Signaling. GSTP1 and SOD1 are mouse monoclonal IgGs from Cell Signaling. TXN, TXNRD1 and PRX1 were included in the PRX pathway cocktail from Abcam. TF is a rabbit monoclonal IgG also from Abcam. The membrane was washed and incubated with horseradish peroxidase (HRP)-conjugated secondary antibody (2 ng/µl) from Cell Signaling. Once the blot was incubated in chemiluminescent substrate, signals were captured using Kodak imaging system. Each blot was re-probed for transferrin, as a loading control. The bands on the image were processed and quantified using an open source image processing tool ImageJ 1.48 V (http://imagej.nih.gov/ij) software. The relative fold changes for these proteins and statistical significance computed using student’s t-Test. In the main figures, the majority of blot images are cropped to represent the targeted proteins. The full length blot images are available in supplemental Fig. S5.

### Immunohistochemistry

Immunohistochemical staining was conducted on paraffin embedded fallopian tissue cross-sections. Briefly Cross-sections were cut 4 µm thick from formalin-fixed paraffin-embedded fallopian tubes. Sections were deparaffinize, rehydrated and antigen retrieval was conducted at 90 °C for 30 min in citrate buffer pH 6.0 with 0.05% Tween. Sections were treated with a peroxidase and alkaline phosphatase blocking reagent (Dako) for 10 min at room temperature then blocked with 2.5% horse serum for 30 minutes. Tissue sections were incubated with anti-MSLN (rabbit monoclonal from Cell Signaling) at a concentration of 52 pg/µl overnight at 4 °C. Sections were then incubated with a peroxidase-conjugated anti–rabbit IgG and visualized with DAB in accordance with manufacturer’s instructions (ImmPress anti-Rabbit and Impact DAB kits from Vector). Cell nuclei were counterstained with hematoxylin for 5 seconds. In contrast, CD45 staining was performed using an automated Bond III staining system (Leica) by the Madigan Army Medical Center anatomical pathology lab (Leica).

### Enzyme-linked immunosorbent assay (ELISA)

Plasma collected from women with hydrosalpinx (n = 9) and healthy controls (n = 9) were processed for ELISA following the manufacturers protocol (R&D Systems). The absorbance readings at 570 nm and for blanks subtracted from 450 nm reading, standard curve was generated and fitted using a four-parameter logistic curve fit (Fig. S4B). Linearity of the assay was assessed using serially diluted plasma containing higher concentration of MSLN per manufacturer recommendation (Fig. S4A).

## Supplementary information

Supplemental Figures

Supplemental Table

## Data Availability

The raw mass spectrometry data sets have been deposited to the proteomeeXchange consortium (ftp://massive.ucsd.edu/MSV000081727) via MassIVE repository.
