# Supplementary material for: The human tubal lavage proteome reveals biological processes that may govern the pathology of hydrosalpinx
Source: Sci Rep. 2019 Jun 20;9:8980. doi: 10.1038/s41598-019-44962-1 (PMC6586608; doi:10.1038/s41598-019-44962-1)
Supplement: Supplementary file 1 — Supplemental Figures [file 41598_2019_44962_MOESM1_ESM.doc]

**The human tubal lavage proteome reveals biological processes that may govern the pathology of hydrosalpinx**

^1*^Elizabeth Yohannes, ^1^Avedis A. Kazanjian, ^2^Morgan E. Lindsay, ^2^Dennis T. Fujii, ^1^Nicholas Ieronimakis, ^2^Gregory E. Chow, ^2^Ronald D. Beesley, ^2^Ryan J. Heitmann, ^1,2^Richard O. Burney


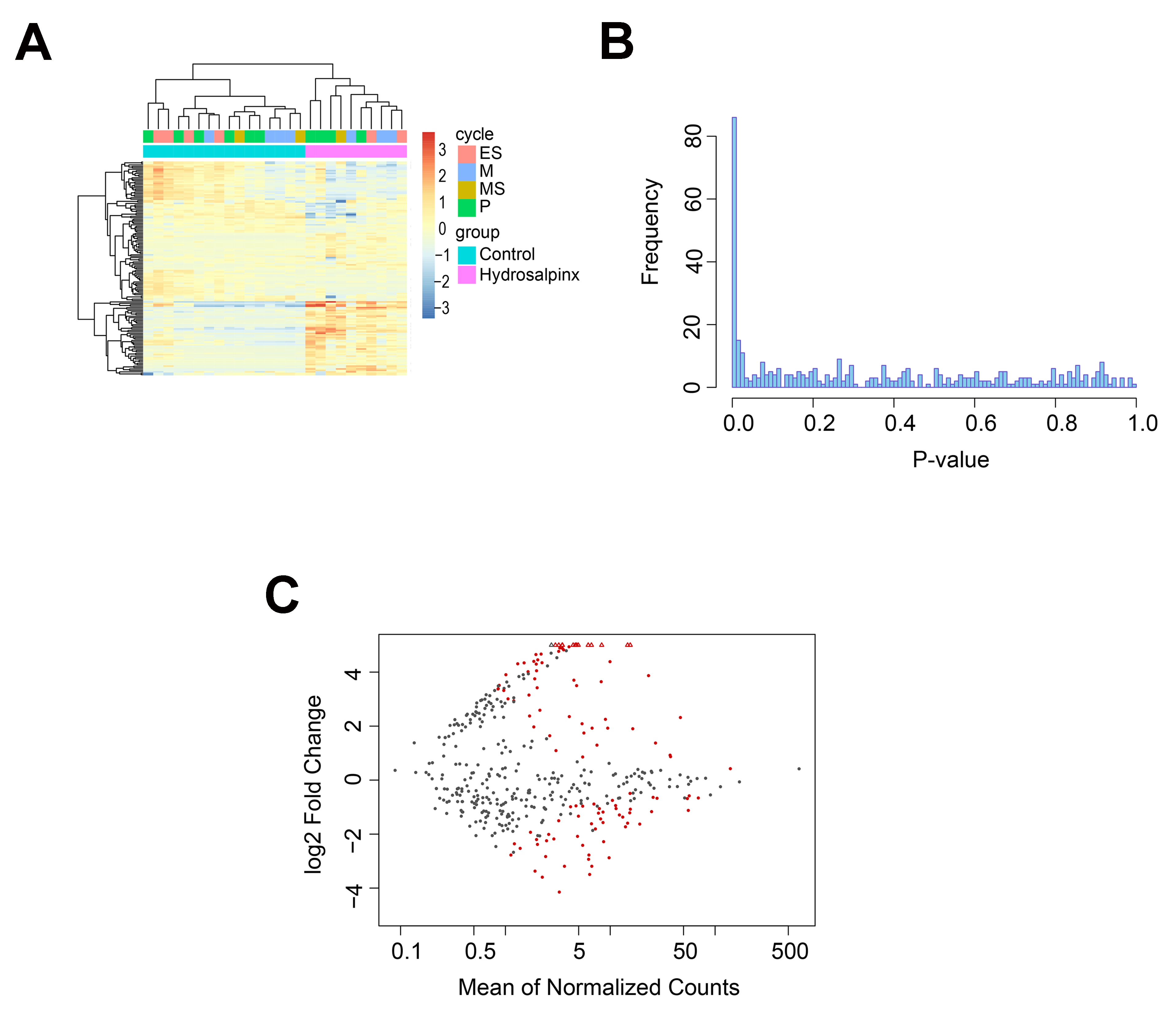


**Figure S1**: Additional high dimensional data summary. **(A)** Hierarchical clustering and heatmap of proteins. In this heatmap proteins and/or samples are grouped together based on protein expression pattern. Samples clustering aligns with experimental factor (hyrosalpinx/lavages). (B) Histogram of p-value from the call to binomial Test. It is evident that there are a set of well-behaved p-values. Flat distribution along the bottom represents null-p-values, which are uniformly distributed between 0 and 1. In this histogram it is also clear that the enrichment of low p-value close zero steams mainly from the differentially expressed proteins in hydrosalpinx relative to healthy lavages along with some null hypotheses that appear at low p-values.

**(C)** The overall protein quantification for the contrast hydrosalpinx verses lavages from healthy control were visualized in log2 fold changes against the mean normalized counts plot.


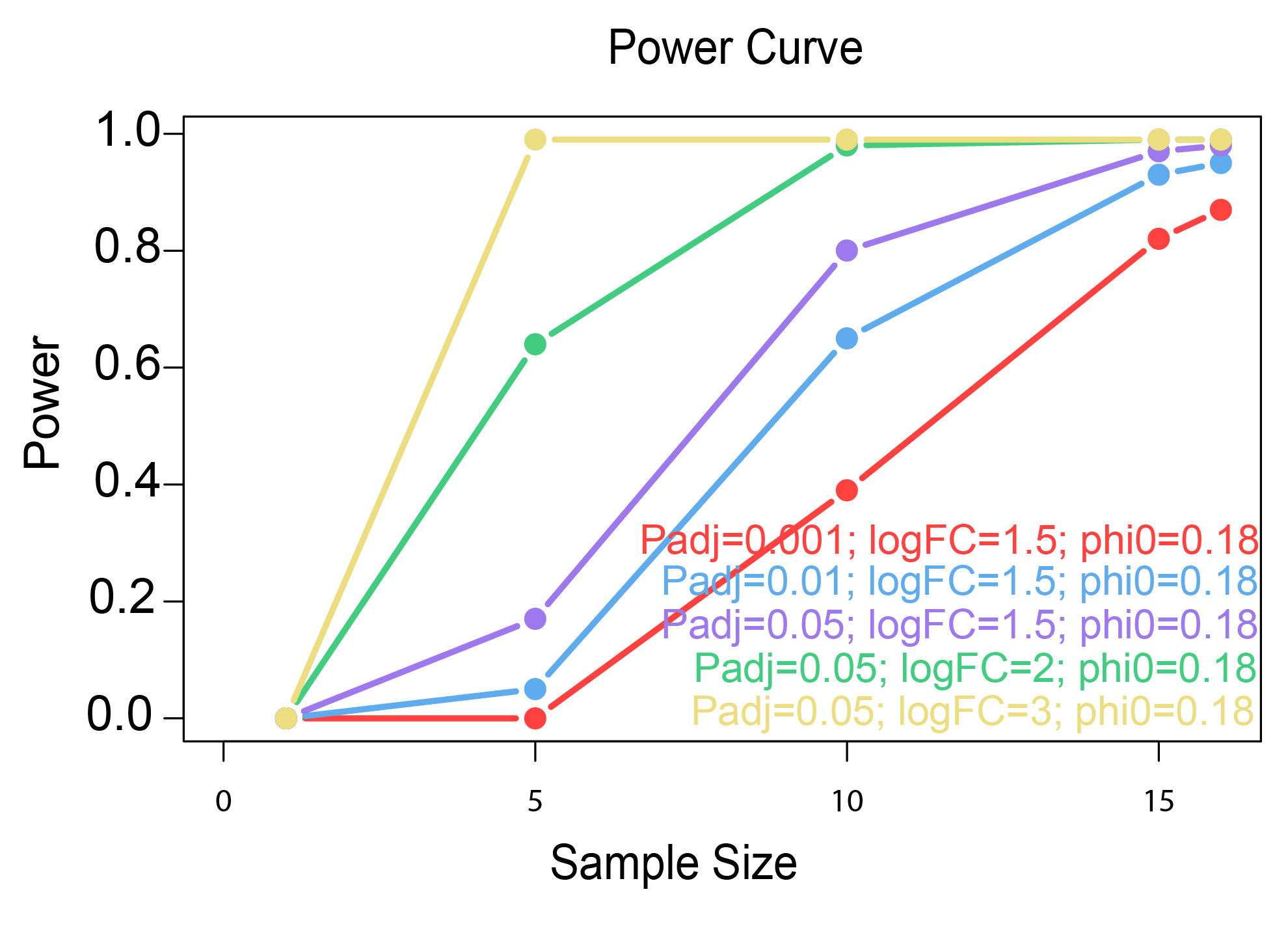


**Figure S2**: The relationship between power and sample size at Padj= 0.001, 0.01 or 0.05 for logFC = 1.5, 2, or 3 in protein expression. It is clear from these curves that with the data median dispersion (dispersion = 0.18 from our pilot study), significance level, and sample size have profound effect on power. As clinical sample numbers were limiting, the power curve at p-adjusted = 0.05 was an appropriate choice for our study and using this curve it was estimated that biological replicates of ten per experimental group results in >= 80% of chance of detecting a 1.5- and above fold change. Thus, we design this study with n=10 for hyrdosapinx and n=16 lavages from normal fallopian tubes.

**Figure S3**: An overview of before and after N-deglycosylation of MSLN and CD59 visualized by gel-shift on SDS PAGE followed by immunoblot analysis. Representative samples from hydrosalpinx and control lavages were treated with PNGase F to release N-linked glycan. De-glycosylated samples were probed with monoclonal anti-MSLN and monoclonal anti-CD59. De-glycosylation of CD59 results a single and sharp band right above 12 Kda indicating a complete de-glycosylation of N-linked glycosylation of CD59. However, the de-glycosylation of MSLN produced three bands that are sharp and distinct compared to a very broad band spanning between 30 Kda and 50 Kda in the original samples.

**Figure S4**: Mesothelin concentration in women with and without hydrosalpinx. **(A)** Linearity of the mesothelin ELISA. A plasma specimen containing 20.56 ng/mL of mesothelin was serially diluted with calibration diluent to produce the expected concentrations of serum mesothelin (x- axis) with the values within the dynamic range of the assay. Points, mean of duplicates. **(B)** Standard curve for human mesothelin: a plot of mean absorbance for each standard with standard error from the mean on the y-axis against the concentration on the x-axis.


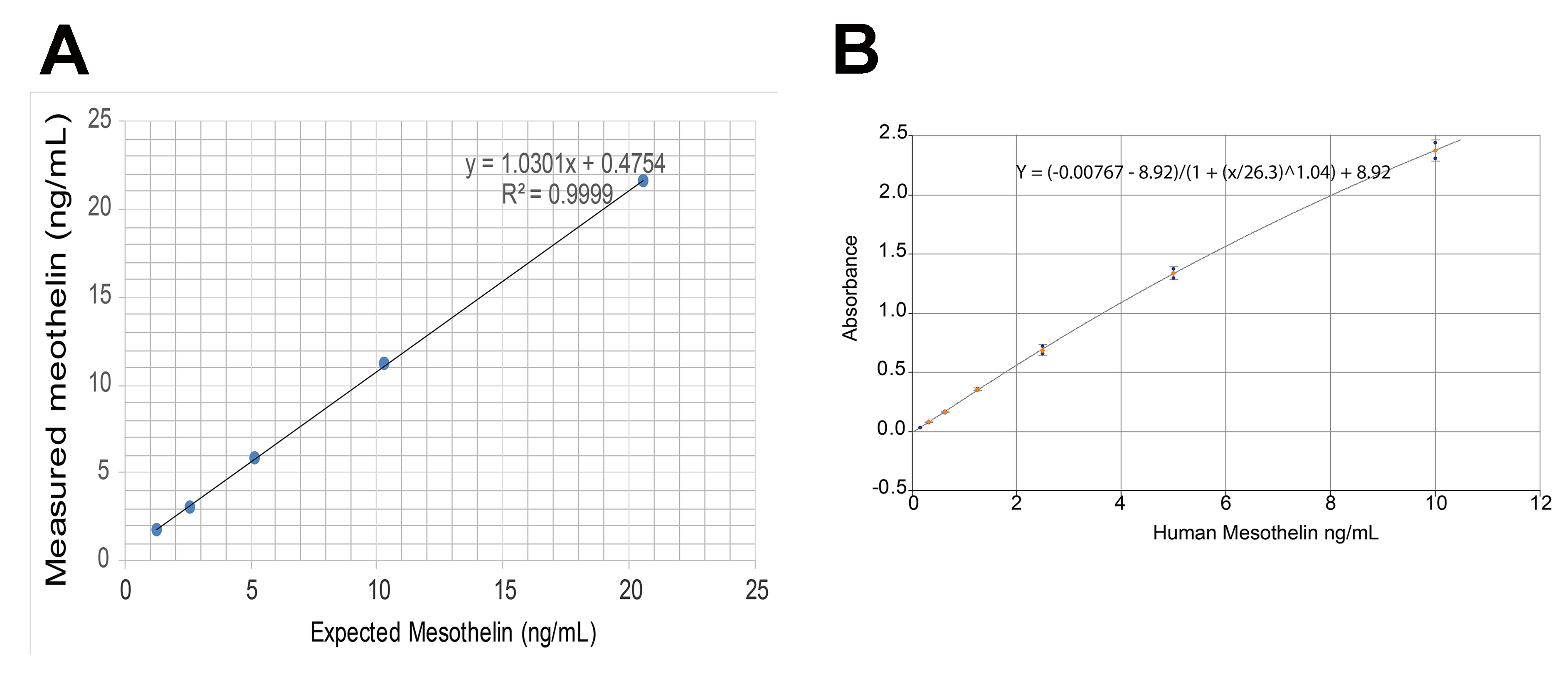


**Figure S5:** Full length blot images related to Figure 4B. (A) Full length images for SOD1 and GSTP1 and TF. B) Full length image for TXNRD1 and TXN with high contrast (left) and low contrast (right). (C) Full length images for MSNL and TF. (D) CD59 with higher and lower magnification along with TF. Each blot in each panel with the exception of blot in panel A has been stripped once for TF analysis as a loading control. Solid arrow denotes the nearest molecular ladder and open arrow indicates the predicted molecular weight of each target.


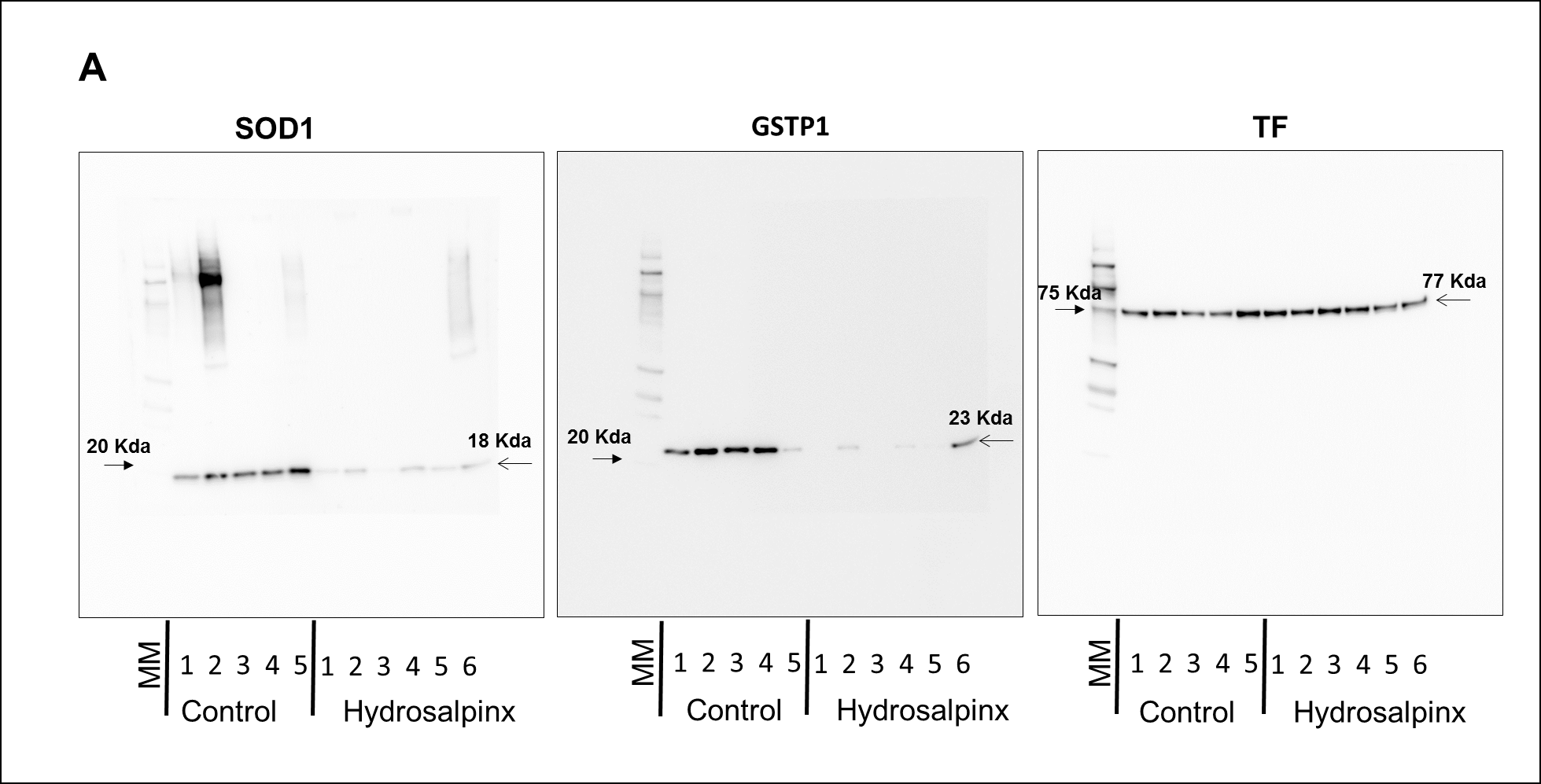


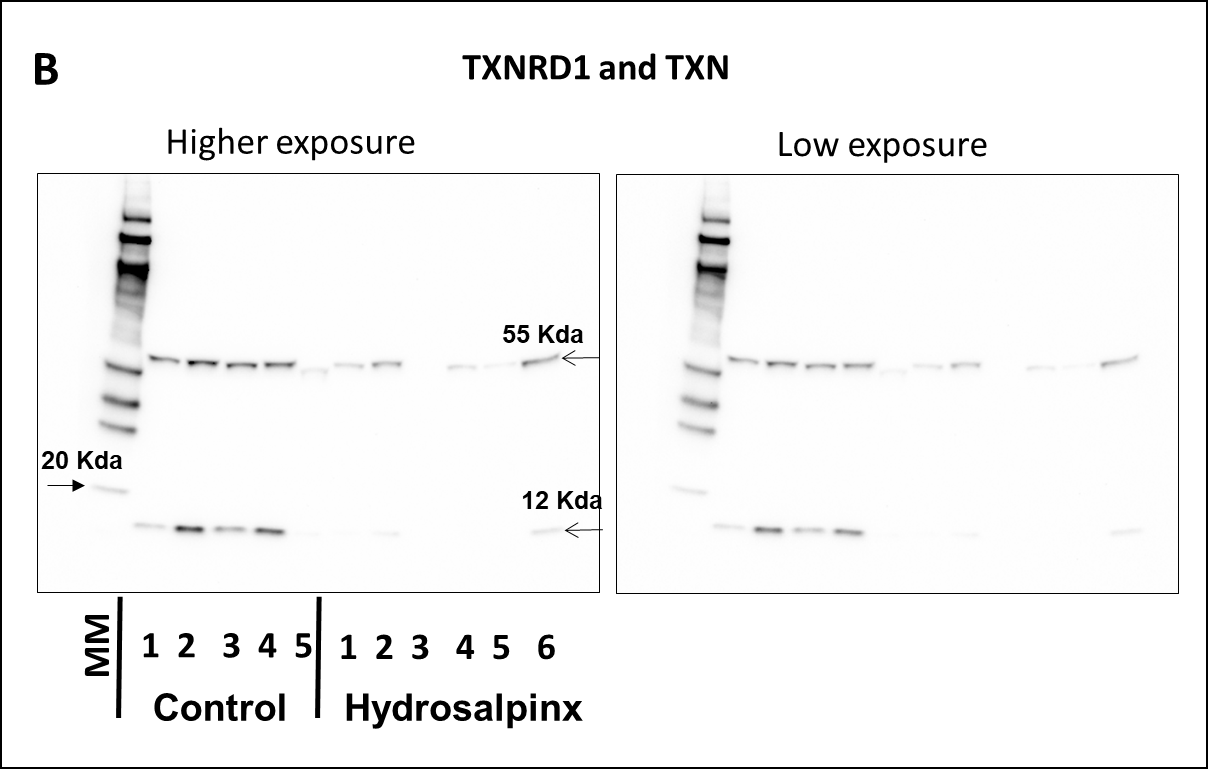


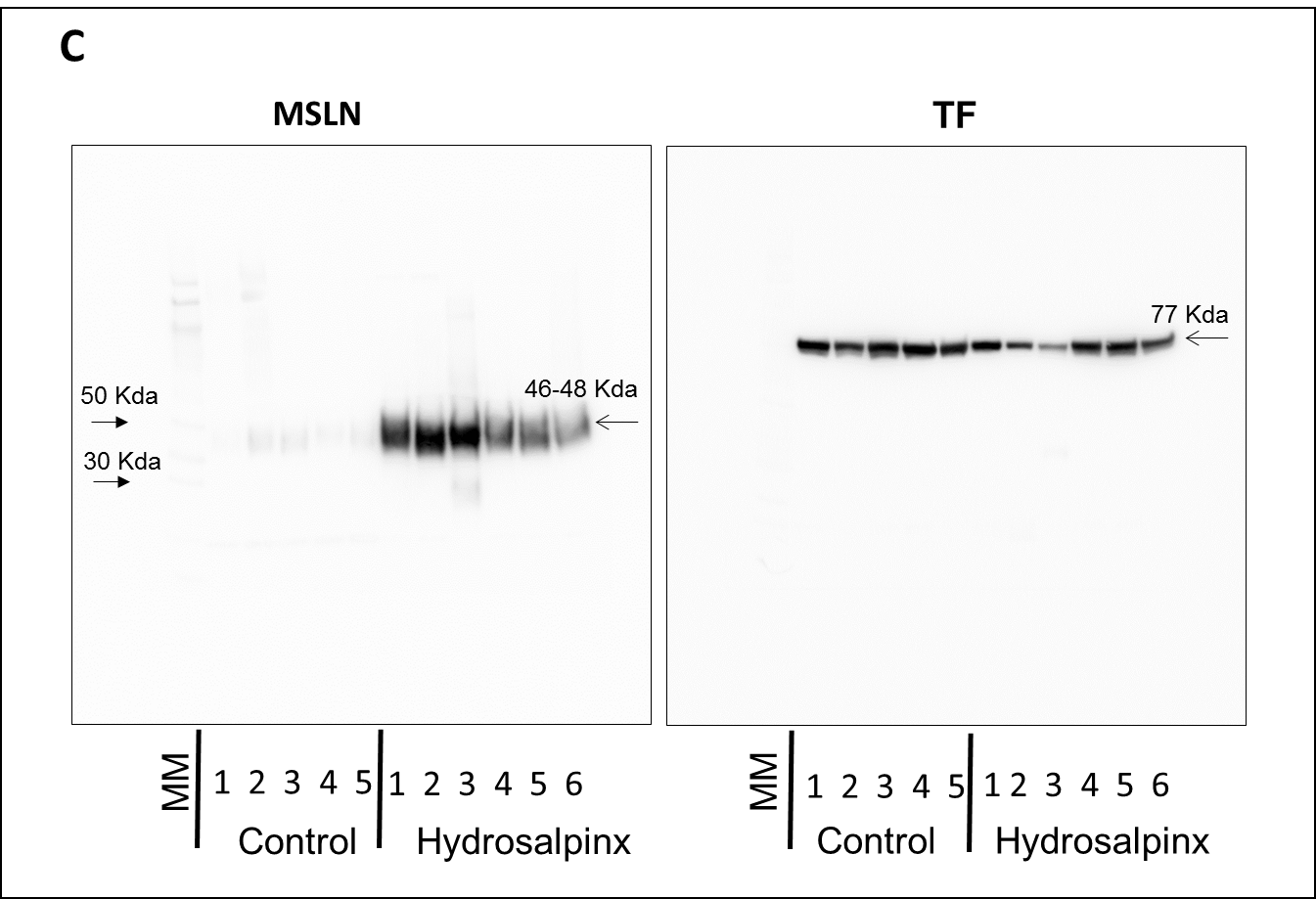


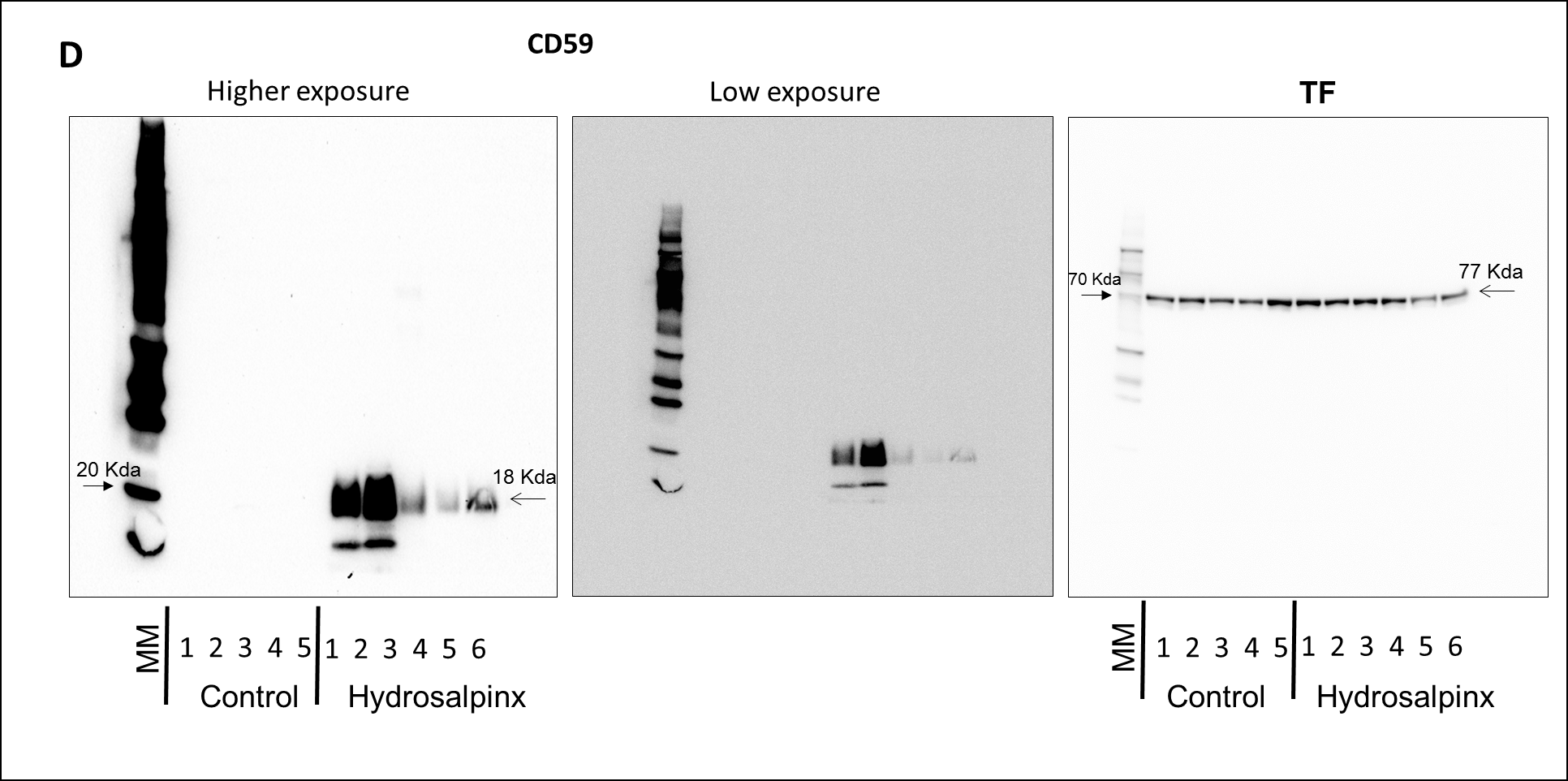


**Table S1**: Patient Information for fallopian tube aspirates (hydrosalpinx) and lavages (from fertile controls undergoing tubal re-anastomosis). A total of 26 independent samples were used for shotgun proteomics. The additional independent control samples were used for western blot verification analysis presented in Figures 4B and C.

|  | Healthy Control | Hydrosalpinx | t-Test  p-value |
| --- | --- | --- | --- |
| Age (years) | 32.1 ± 1.0 2541 | 30.5 ± 2.0 2438 | 0.35 |
| Parity | 2.9 ± 0.3 | 1.4 ± 0.4 | 5.5 x 10^-5^ |
| BMI | 26.1 ± 1.0 | 24.5 ± 1.6 | 0.28 |
| Serum Estradiol (pg/mL) | 140.2 ± 24.2 | 121.7 ± 19.8 | 0.26 |
| Serum Progesterone (ng/mL) | 3.9 ± 1.1 | 5.4 ± 1.3 | 0.38 |
| **Cycle Phases, n patients:** |  |  |  |
| Menstrual (M) | 6 | 3 |  |
| Proliferative (P) | 6 | 4 |  |
| Early-Secretory (ES) | 4 | 2 |  |
| Mid-Secretory (MS) | 4 | 1 |  |
| **Chi-Square *Test*** | 0.896 | |  |

**Table S2**: Differential proteome profile of tubal fluids from subjects with hydrosalpinx relative to lavages from healthy fertile donors.

| Accession | Protein identity | Log 2 Fold change | P-Adj | Biological Process |
| --- | --- | --- | --- | --- |
| Q08380 | Galectin-3-binding protein OS=Homo sapiens GN=LGALS3BP PE=1 SV=1 - [LG3BP_HUMAN] | 6.38 | 9.79E-20 | Platelet degranulation, cell defense response, cell adhesion, activation of neutrophils & granulocytes |
| P10909 | Clusterin OS=Homo sapiens GN=CLU PE=1 SV=1 - [CLUS_HUMAN] | 2.3 | 5.67E-15 | Complement activation, cell death, innate immune response, recruitment of phagocytes |
| P01833 | Polymeric immunoglobulin receptor OS=Homo sapiens GN=PIGR PE=1 SV=4 - [PIGR_HUMAN] | 8.32 | 4.12E-11 | Immune Cell trafficking, immunoglobulin transcytosis in epithelial cells |
| Q13421 | Mesothelin OS=Homo sapiens GN=MSLN PE=1 SV=2 - [MSLN_HUMAN] | 4.2 | 1.29E-09 | Cell adhesion |
| P80188 | Neutrophil gelatinase-associated lipocalin OS=Homo sapiens GN=LCN2 PE=1 SV=2 - [NGAL_HUMAN] | 7.36 | 2.71E-09 | Innate immune response, activation of macrophages, recruitment of neutrophils, chemotaxis of neutrophils, ion transport, inflammation of mucosa |
| P02750 | Leucine-rich alpha-2-glycoprotein OS=Homo sapiens GN=LRG1 PE=1 SV=2 - [A2GL_HUMAN] | 2.23 | 4.44E-09 | Positive regulator of endothelial cell proliferation, positive regulation of agiogenesis |
| P10451 | Osteopontin OS=Homo sapiens GN=SPP1 PE=1 SV=1 - [OSTP_HUMAN] | 7.67 | 4.44E-09 | Inflammatory response, Osteoblast differentiation, cell adhesion, recruitment & accumulation of macrophages |
| P04745 | Alpha-amylase 1 OS=Homo sapiens GN=AMY1A PE=1 SV=2 - [AMY1_HUMAN] | 5.78 | 4.85E-09 | Cabohydrate metabolic process |
| Q14508 | WAP four-disulfide core domain protein 2 OS=Homo sapiens GN=WFDC2 PE=1 SV=2 - [WFDC2_HUMAN] | 5.1 | 8.08E-09 | Proteolysis |
| Q8WXI7 | Mucin-16 OS=Homo sapiens GN=MUC16 PE=1 SV=3 - [MUC16_HUMAN] | 7.55 | 1.51E-08 | Cell adhesion |
| P14384 | Carboxypeptidase M OS=Homo sapiens GN=CPM PE=1 SV=2 - [CBPM_HUMAN] | 7.25 | 2.23E-08 | peptide metabolic process, anatomical structure morphogenesis |
| P05155 | Plasma protease C1 inhibitor OS=Homo sapiens GN=SERPING1 PE=1 SV=2 - [IC1_HUMAN] | 1.89 | 5.09E-08 | complement activation, classical pathway, innate immune response, accumulation of granulocytes |
| P12109 | Collagen alpha-1(VI) chain OS=Homo sapiens GN=COL6A1 PE=1 SV=3 - [CO6A1_HUMAN] | 6.84 | 1.75E-07 | cell adhesion, extracellular matrix organization, osteoblast differentiation, |
| P23142 | Fibulin-1 OS=Homo sapiens GN=FBLN1 PE=1 SV=4 - [FBLN1_HUMAN] | 7.39 | 2.08E-07 | integrin-mediated signaling pathway, embryo implantation, extracellular matrix organization |
| P05156 | Complement factor I OS=Homo sapiens GN=CFI PE=1 SV=2 - [CFAI_HUMAN] | 1.91 | 2.69E-07 | complement activation, classical pathway, regulation of complement activation, innate immune response, |
| O00391 | Sulfhydryl oxidase 1 OS=Homo sapiens GN=QSOX1 PE=1 SV=3 - [QSOX1_HUMAN] | 4.97 | 3.19E-07 | negative regulation of macroautophagy, cell redox homeostasis, oxidation-reduction process, |
| Q14515 | SPARC-like protein 1 OS=Homo sapiens GN=SPARCL1 PE=1 SV=2 - [SPRL1_HUMAN] | 4.33 | 7.85E-07 | signal transduction, anatomical structure development |
| Q86WI1 | Fibrocystin-L OS=Homo sapiens GN=PKHD1L1 PE=2 SV=2 - [PKHL1_HUMAN] | 6.27 | 9.12E-07 | immune response |
| Q16270 | Insulin-like growth factor-binding protein 7 OS=Homo sapiens GN=IGFBP7 PE=1 SV=1 - [IBP7_HUMAN] | 6.86 | 1.08E-06 | regulation of cell growth, cell adhesion, embryo implantation, |
| O00592 | Podocalyxin OS=Homo sapiens GN=PODXL PE=1 SV=2 - [PODXL_HUMAN] | 6.09 | 1.48E-06 | negative regulation of cell adhesion, cell migration, epithelial tube formation, |
| P12821 | Angiotensin-converting enzyme OS=Homo sapiens GN=ACE PE=1 SV=1 - [ACE_HUMAN] | 6.35 | 1.57E-06 | inflammatory response, activation of macrophages, neutrophil mediated immunity, activation of antigen presenting cells, blood vessel remodeling |
| P00747 | Plasminogen OS=Homo sapiens GN=PLG PE=1 SV=2 - [PLMN_HUMAN] | -1.71 | 1.57E-06 | platelet degranulation, accumulation of granulocytes, movement of monocytes extracellular matrix disassembly, tissue remodeling |
| P61769 | Beta-2-microglobulin OS=Homo sapiens GN=B2M PE=1 SV=1 - [B2MG_HUMAN] | 5.22 | 3.30E-06 | positive regulation of T cell mediated cytotoxicity, antigen processing and presentation of peptide antigen via MHC class I, negative regulation of neuron projection development, immune response |
| P15941 | Mucin-1 OS=Homo sapiens GN=MUC1 PE=1 SV=3 - [MUC1_HUMAN] | 4.2 | 3.78E-06 | response to hypoxia, DNA damage response, signal transduction |
| O43490 | Prominin-1 OS=Homo sapiens GN=PROM1 PE=1 SV=1 - [PROM1_HUMAN] | 6.3 | 4.77E-06 | retina layer formation, positive regulation of nephron tubule epithelial cell differentiation, atopic dermatitis |
| P02788 | Lactotransferrin OS=Homo sapiens GN=LTF PE=1 SV=6 - [TRFL_HUMAN] | 6.49 | 5.09E-06 | innate immune response in mucosa, ion transport, activation of neutrophils & macrophages |
| P36222 | Chitinase-3-like protein 1 OS=Homo sapiens GN=CHI3L1 PE=1 SV=2 - [CH3L1_HUMAN] | 5.76 | 7.18E-06 | activation of phagocytes, recruitment of neutrophils, carbohydrate metabolic process, chitin catabolic |
| Q12889 | Oviduct-specific glycoprotein OS=Homo sapiens GN=OVGP1 PE=2 SV=1 - [OVGP1_HUMAN] | 3.49 | 7.23E-06 | negative regulation of binding of sperm to zona pellucida |
| Q9BW30 | Tubulin polymerization-promoting protein family member 3 OS=Homo sapiens GN=TPPP3 PE=1 SV=1 - [TPPP3_HUMAN] | -2.78 | 8.55E-06 | microtubule bundle formation |
| P09211 | Glutathione S-transferase P OS=Homo sapiens GN=GSTP1 PE=1 SV=2 - [GSTP1_HUMAN] | -2.24 | 1.41E-05 | response to reactive oxygen species, negative regulation of acute inflammatory response |
| P13671 | Complement component C6 OS=Homo sapiens GN=C6 PE=1 SV=3 - [CO6_HUMAN] | 3.34 | 1.43E-05 | complement activation |
| Q13228 | Selenium-binding protein 1 OS=Homo sapiens GN=SELENBP1 PE=1 SV=2 - [SBP1_HUMAN] | -3.39 | 1.51E-05 | protein transport, allergic pulmonary eosinophilia, |
| P06396 | Gelsolin OS=Homo sapiens GN=GSN PE=1 SV=1 - [GELS_HUMAN] | 1.66 | 1.55E-05 | chemotaxis of leukocytes, phagocytosis, immune response of antigen presenting cells |
| Q16651 | Prostasin OS=Homo sapiens GN=PRSS8 PE=1 SV=1 - [PRSS8_HUMAN] | 5.98 | 1.88E-05 | proteolysis |
| Q13938 | Calcyphosin OS=Homo sapiens GN=CAPS PE=1 SV=1 - [CAYP1_HUMAN] | -1.58 | 1.98E-05 | intracellular signal transduction |
| O00468 | Agrin OS=Homo sapiens GN=AGRN PE=1 SV=5 - [AGRIN_HUMAN] | 6.27 | 2.00E-05 | glycosaminoglycan biosynthetic process, activation of leukocytes |
| P13987 | CD59 glycoprotein OS=Homo sapiens GN=CD59 PE=1 SV=1 - [CD59_HUMAN] | 4.99 | 2.56E-05 | regulation of complement activation, immune response, activation of mononuclear leukocytes |
| Q9NQ79 | Cartilage acidic protein 1 OS=Homo sapiens GN=CRTAC1 PE=1 SV=2 - [CRAC1_HUMAN] | 5.35 | 5.95E-05 | axonal fasciculation |
| P00568 | Adenylate kinase isoenzyme 1 OS=Homo sapiens GN=AK1 PE=1 SV=3 - [KAD1_HUMAN] | -5.77 | 7.20E-05 | nucleobase-containing compound metabolic process |
| P08294 | Extracellular superoxide dismutase [Cu-Zn] OS=Homo sapiens GN=SOD3 PE=1 SV=2 - [SODE_HUMAN] | 5.1 | 7.47E-05 | response to reactive oxygen species, cellular infiltration by macrophages |
| P01034 | Cystatin-C OS=Homo sapiens GN=CST3 PE=1 SV=1 - [CYTC_HUMAN] | 3.66 | 1.15E-04 | chemotaxis of leukocytes, accumulation of neutrophils, response to hypoxia, apoptotic process |
| P02792 | Ferritin light chain OS=Homo sapiens GN=FTL PE=1 SV=2 - [FRIL_HUMAN] | 4.96 | 1.42E-04 | iron ion transport, inflammation, synovitis |
| Q8N2S1 | Latent-transforming growth factor beta-binding protein 4 OS=Homo sapiens GN=LTBP4 PE=1 SV=2 - [LTBP4_HUMAN] | 5.81 | 1.57E-04 | regulation of cell growth |
| P04083 | Annexin A1 OS=Homo sapiens GN=ANXA1 PE=1 SV=2 - [ANXA1_HUMAN] | -3.12 | 1.60E-04 | neutrophil homeostasis, adaptive immune response, monocyte chemotaxis, migration & accumulation of neutrophils, adhesion of neutrophil, inflammation of mucosa |
| P09466 | Glycodelin OS=Homo sapiens GN=PAEP PE=1 SV=2 - [PAEP_HUMAN] | 6.37 | 1.66E-04 | apoptotic process, regulation of interleukin-13 secretion, positive regulation of interleukin-6 secretion |
| P04075 | Fructose-bisphosphate aldolase A OS=Homo sapiens GN=ALDOA PE=1 SV=2 - [ALDOA_HUMAN] | -2.71 | 1.80E-04 | glycolytic process, tissue necrosis, inflammation of body cavity, body organ & joints |
| P15291 | Beta-1,4-galactosyltransferase 1 OS=Homo sapiens GN=B4GALT1 PE=1 SV=5 - [B4GT1_HUMAN] | 5.53 | 1.91E-04 | acute inflammatory response, chemotaxis of leukocytes, binding of neutrophils & professional phagocytic cells, epithelial cell development |
| P08123 | Collagen alpha-2(I) chain OS=Homo sapiens GN=COL1A2 PE=1 SV=7 - [CO1A2_HUMAN] | 3.25 | 2.25E-04 | skeletal system development, extracellular matrix organization, colitis |
| P54108 | Cysteine-rich secretory protein 3 OS=Homo sapiens GN=CRISP3 PE=1 SV=1 - [CRIS3_HUMAN] | 3.46 | 2.25E-04 | defense response, innate immune response |
| P08185 | Corticosteroid-binding globulin OS=Homo sapiens GN=SERPINA6 PE=1 SV=1 - [CBG_HUMAN] | 5.27 | 2.27E-04 | glucocorticoid metabolic process |
| P30044 | Peroxiredoxin-5, mitochondrial OS=Homo sapiens GN=PRDX5 PE=1 SV=4 - [PRDX5_HUMAN] | -5.31 | 3.37E-04 | response to reactive oxygen species, hydrogen peroxide catabolic process, inflammatory response |
| P30086 | Phosphatidylethanolamine-binding protein 1 OS=Homo sapiens GN=PEBP1 PE=1 SV=3 - [PEBP1_HUMAN] | -2.85 | 3.71E-04 | negative regulation of endopeptidase activity |
| P08758 | Annexin A5 OS=Homo sapiens GN=ANXA5 PE=1 SV=2 - [ANXA5_HUMAN] | -2.34 | 5.81E-04 | immune response leukocytes, phagocytosis, signal transduction, apoptotic process |
| P08603 | Complement factor H OS=Homo sapiens GN=CFH PE=1 SV=4 - [CFAH_HUMAN] | -1.62 | 6.22E-04 | regulation of complement activation, immune response of leukocytes, binding of neutrophils, phagocytosis |
| P05787 | Keratin, type II cytoskeletal 8 OS=Homo sapiens GN=KRT8 PE=1 SV=7 - [K2C8_HUMAN] | -5.07 | 7.21E-04 | tumor necrosis factor-mediated signaling pathway, cell differentiation involved in embryonic placenta development |
| P01024 | Complement C3 OS=Homo sapiens GN=C3 PE=1 SV=2 - [CO3_HUMAN] | 1.62 | 9.62E-04 | complement activation, inflammatory response |
| P61916 | Epididymal secretory protein E1 OS=Homo sapiens GN=NPC2 PE=1 SV=1 - [NPC2_HUMAN] | 5.12 | 0.001 | cholesterol metabolic process |
| P15328 | Folate receptor alpha OS=Homo sapiens GN=FOLR1 PE=1 SV=3 - [FOLR1_HUMAN] | 5.17 | 0.001 | folic acid transport, cellular response to folic acid |
| P39060 | Collagen alpha-1(XVIII) chain OS=Homo sapiens GN=COL18A1 PE=1 SV=5 - [COIA1_HUMAN] | 5.45 | 0.002 | endothelial cell morphogenesis, extracellular matrix organization, accumulation of leukocytes |
| P21291 | Cysteine and glycine-rich protein 1 OS=Homo sapiens GN=CSRP1 PE=1 SV=3 - [CSRP1_HUMAN] | -4.5 | 0.002 | platelet aggregation |
| P61626 | Lysozyme C OS=Homo sapiens GN=LYZ PE=1 SV=1 - [LYSC_HUMAN] | 4.49 | 0.002 | inflammatory response, metabolic process, antibacterial response |
| P26038 | Moesin OS=Homo sapiens GN=MSN PE=1 SV=3 - [MOES_HUMAN] | 1.96 | 0.002 | movement of cell or subcellular component, leukocyte cell-cell adhesion |
| P62937 | Peptidyl-prolyl cis-trans isomerase A OS=Homo sapiens GN=PPIA PE=1 SV=2 - [PPIA_HUMAN] | -2.07 | 0.002 | protein peptidyl-prolyl isomerization, chemotaxis of monocytes |
| P13611 | Versican core protein OS=Homo sapiens GN=VCAN PE=1 SV=3 - [CSPG2_HUMAN] | 4.7 | 0.002 | skeletal system development |
| P08697 | Alpha-2-antiplasmin OS=Homo sapiens GN=SERPINF2 PE=1 SV=3 - [A2AP_HUMAN] | -1.96 | 0.003 | platelet degranulation, acute-phase response |
| P08174 | Complement decay-accelerating factor OS=Homo sapiens GN=CD55 PE=1 SV=4 - [DAF_HUMAN] | 5.16 | 0.003 | complement activation, innate immune response |
| P02649 | Apolipoprotein E OS=Homo sapiens GN=APOE PE=1 SV=1 - [APOE_HUMAN] | 1.87 | 0.004 | cholesterol metabolic process, negative regulation of inflammatory response, response to reactive oxygen species |
| P51884 | Lumican OS=Homo sapiens GN=LUM PE=1 SV=2 - [LUM_HUMAN] | 3.31 | 0.004 | organismal Injury, axonogenesis |
| P01033 | Metalloproteinase inhibitor 1 OS=Homo sapiens GN=TIMP1 PE=1 SV=1 - [TIMP1_HUMAN] | 4.4 | 0.004 | cell activation, platelet degranulation, accumulation of macrophages |
| Q96C23 | Aldose 1-epimerase OS=Homo sapiens GN=GALM PE=1 SV=1 - [GALM_HUMAN] | -4.47 | 0.005 | carbohydrate metabolic process, chitin catabolic |
| P05186 | Alkaline phosphatase, tissue-nonspecific isozyme OS=Homo sapiens GN=ALPL PE=1 SV=4 - [PPBT_HUMAN] | 4.91 | 0.006 | skeletal system development |
| P03973 | Antileukoproteinase OS=Homo sapiens GN=SLPI PE=1 SV=2 - [SLPI_HUMAN] | 2.45 | 0.006 | innate immune response, antibacterial humoral response |
| P49747 | Cartilage oligomeric matrix protein OS=Homo sapiens GN=COMP PE=1 SV=2 - [COMP_HUMAN] | 5.22 | 0.006 | skeletal system development, apoptotic process |
| P12277 | Creatine kinase B-type OS=Homo sapiens GN=CKB PE=1 SV=1 - [KCRB_HUMAN] | -3.21 | 0.006 | creatine metabolic process, immune response of cells, phagocytosis |
| Q9HC84 | Mucin-5B OS=Homo sapiens GN=MUC5B PE=1 SV=3 - [MUC5B_HUMAN] | 4.7 | 0.006 | regulation of macrophage activation, antibacterial response, O-glycan processing |
| P54652 | Heat shock-related 70 kDa protein 2 OS=Homo sapiens GN=HSPA2 PE=1 SV=1 - [HSP72_HUMAN] | -1.6 | 0.008 | response to unfolded protein, regulation of cell death |
| P04792 | Heat shock protein beta-1 OS=Homo sapiens GN=HSPB1 PE=1 SV=2 - [HSPB1_HUMAN] | -1.73 | 0.009 | oxidative stress-induced intrinsic apoptotic signaling pathway, response to unfolded protein, cell movement |
| P41222 | Prostaglandin-H2 D-isomerase OS=Homo sapiens GN=PTGDS PE=1 SV=1 - [PTGDS_HUMAN] | 4.69 | 0.009 | prostaglandin biosynthetic process, accumulation of granulocytes |
| P11142 | Heat shock cognate 71 kDa protein OS=Homo sapiens GN=HSPA8 PE=1 SV=1 - [HSP7C_HUMAN] | -1.54 | 0.011 | response to unfolded protein |
| P02794 | Ferritin heavy chain OS=Homo sapiens GN=FTH1 PE=1 SV=2 - [FRIH_HUMAN] | 4.51 | 0.014 | iron ion transport, immune response, oxidation-reduction process |
| Q06828 | Fibromodulin OS=Homo sapiens GN=FMOD PE=1 SV=2 - [FMOD_HUMAN] | 4.36 | 0.014 | complement activation |
| Q8NBJ4 | Golgi membrane protein 1 OS=Homo sapiens GN=GOLM1 PE=1 SV=1 - [GOLM1_HUMAN] | 4.23 | 0.014 | nucleus organization |
| Q7L266 | Isoaspartyl peptidase/L-asparaginase OS=Homo sapiens GN=ASRGL1 PE=1 SV=2 - [ASGL1_HUMAN] | -3.11 | 0.014 | proteolysis |
| P08571 | Monocyte differentiation antigen CD14 OS=Homo sapiens GN=CD14 PE=1 SV=2 - [CD14_HUMAN] | 3.28 | 0.014 | apoptotic process, inflammatory response, activation of leukocytes, production of ROS |
| P55058 | Phospholipid transfer protein OS=Homo sapiens GN=PLTP PE=1 SV=1 - [PLTP_HUMAN] | 4.29 | 0.014 | lipid transport, inflammatory response, binding of neutrophils, sperm motility |
| P35241 | Radixin OS=Homo sapiens GN=RDX PE=1 SV=1 - [RADI_HUMAN] | 1.56 | 0.014 | regulation of cell shape |
| P60174 | Triosephosphate isomerase OS=Homo sapiens GN=TPI1 PE=1 SV=3 - [TPIS_HUMAN] | -1.5 | 0.014 | gluconeogenesis, canonical glycolysis |
| P20061 | Transcobalamin-1 OS=Homo sapiens GN=TCN1 PE=1 SV=2 - [TCO1_HUMAN] | 4.79 | 0.015 | cobalt ion transport |
| P0DMV9 | Heat shock 70 kDa protein 1B OS=Homo sapiens GN=HSPA1B PE=1 SV=1 - [HS71B_HUMAN] | -1.5 | 0.016 | regulation of cell death, cellular response to oxidative stress |
| P09871 | Complement C1s subcomponent OS=Homo sapiens GN=C1S PE=1 SV=1 - [C1S_HUMAN] | 1.87 | 0.017 | complement activation, innate immune response |
| P23526 | Adenosylhomocysteinase OS=Homo sapiens GN=AHCY PE=1 SV=4 - [SAHH_HUMAN] | -4 | 0.018 | sulfur amino acid metabolic process, chronic inflammatory response to antigenic stimulus, response to hypoxia |
| O43866 | CD5 antigen-like OS=Homo sapiens GN=CD5L PE=1 SV=1 - [CD5L_HUMAN] | -2.75 | 0.019 | inflammatory response, apoptotic process, recruitment of phagocytes |
| P0C0L4 | Complement C4-A OS=Homo sapiens GN=C4A PE=1 SV=2 - [CO4A_HUMAN] | 1.59 | 0.019 | complement activation, inflammatory response, activation of leukocytes |
| P07900 | Heat shock protein HSP 90-alpha OS=Homo sapiens GN=HSP90AA1 PE=1 SV=5 - [HS90A_HUMAN] | -1.69 | 0.019 | protein folding, immune response of antigen presenting cells |
| P09429 | High mobility group protein B1 OS=Homo sapiens GN=HMGB1 PE=1 SV=3 - [HMGB1_HUMAN] | -4.29 | 0.019 | activation of innate immune response, regulation of tolerance induction |
| P07225 | Vitamin K-dependent protein S OS=Homo sapiens GN=PROS1 PE=1 SV=1 - [PROS_HUMAN] | 4.46 | 0.019 | leukocyte migration, regulation of complement activation |
| Q8IZP2 | Putative protein FAM10A4 OS=Homo sapiens GN=ST13P4 PE=5 SV=1 - [ST134_HUMAN] | -3.75 | 0.021 | NA |
| P00441 | Superoxide dismutase [Cu-Zn] OS=Homo sapiens GN=SOD1 PE=1 SV=2 - [SODC_HUMAN] | -1.52 | 0.022 | response to reactive oxygen species, ovarian follicle development |
| P0C0L5 | Complement C4-B OS=Homo sapiens GN=C4B PE=1 SV=2 - [CO4B_HUMAN] | 0.86 (NS) | 0.023 | complement activation, inflammatory response |
| Q14103 | Heterogeneous nuclear ribonucleoprotein D0 OS=Homo sapiens GN=HNRNPD PE=1 SV=1 - [HNRPD_HUMAN] | -3.87 | 0.023 | mRNA splicing, via spliceosome |
| Q99497 | Protein DJ-1 OS=Homo sapiens GN=PARK7 PE=1 SV=2 - [PARK7_HUMAN] | -2.29 | 0.027 | inflammatory response, cellular response to oxidative stress, hydrogen peroxide metabolic process, protein stabilization |
| P06702 | Protein S100-A9 OS=Homo sapiens GN=S100A9 PE=1 SV=1 - [S10A9_HUMAN] | 3.28 | 0.027 | leukocyte migration involved in inflammatory response, neutrophil chemotaxis, accumulation of macrophages |
| P43353 | Aldehyde dehydrogenase family 3 member B1 OS=Homo sapiens GN=ALDH3B1 PE=1 SV=1 - [AL3B1_HUMAN] | 4.28 | 0.028 | alcohol metabolic process, cell death, necrosis, cellular response to oxidative stress |
| P00736 | Complement C1r subcomponent OS=Homo sapiens GN=C1R PE=1 SV=2 - [C1R_HUMAN] | 2.87 | 0.028 | complement activation, immune response |
| P04406 | Glyceraldehyde-3-phosphate dehydrogenase OS=Homo sapiens GN=GAPDH PE=1 SV=3 - [G3P_HUMAN] | -1.94 | 0.028 | glycolytic process, apoptotic process, DNA repair |
| P30041 | Peroxiredoxin-6 OS=Homo sapiens GN=PRDX6 PE=1 SV=3 - [PRDX6_HUMAN] | -2.06 | 0.029 | response to reactive oxygen species, hydrogen peroxide catabolic process |
| P06454 | Prothymosin alpha OS=Homo sapiens GN=PTMA PE=1 SV=2 - [PTMA_HUMAN] | -2.95 | 0.029 | transcription |
| P52943 | Cysteine-rich protein 2 OS=Homo sapiens GN=CRIP2 PE=1 SV=1 - [CRIP2_HUMAN] | -3.17 | 0.032 | positive regulation of cell proliferation |
| P05154 | Plasma serine protease inhibitor OS=Homo sapiens GN=SERPINA5 PE=1 SV=3 - [IPSP_HUMAN] | 2.79 | 0.038 | lipid transport |
| O00560 | Syntenin-1 OS=Homo sapiens GN=SDCBP PE=1 SV=1 - [SDCB1_HUMAN] | 4.24 | 0.039 | negative regulation of receptor internalization |
| P10599 | Thioredoxin OS=Homo sapiens GN=TXN PE=1 SV=3 - [THIO_HUMAN] | -2.33 | 0.039 | response to reactive oxygen species, hydrogen peroxide catabolic process |
| Q7Z7G0 | Target of Nesh-SH3 OS=Homo sapiens GN=ABI3BP PE=1 SV=1 - [TARSH_HUMAN] | 4.38 | 0.04 | extracellular matrix organization |
| P05109 | Protein S100-A8 OS=Homo sapiens GN=S100A8 PE=1 SV=1 - [S10A8_HUMAN] | 3.94 | 0.046 | leukocyte migration involved in inflammatory response, neutrophil chemotaxis, chronic inflammatory response |
| P29401 | Transketolase OS=Homo sapiens GN=TKT PE=1 SV=3 - [TKT_HUMAN] | -2.28 | 0.046 | xylulose biosynthetic process |
| P22626 | Heterogeneous nuclear ribonucleoproteins A2/B1 OS=Homo sapiens GN=HNRNPA2B1 PE=1 SV=2 - [ROA2_HUMAN] | -3.75 | 0.047 | transcription from RNA polymerase II promoter |
| P27169 | Serum paraoxonase/arylesterase 1 OS=Homo sapiens GN=PON1 PE=1 SV=3 - [PON1_HUMAN] | -1.94 | 0.048 | response to toxic substance, response to reactive oxygen species |
